# Multiplex detection of seven transgenes for human gene doping analysis

**DOI:** 10.1038/s41598-025-06677-4

**Published:** 2025-06-20

**Authors:** Nana Naumann, Carolin Do, Caren Vollmert, Maroje Krajina, Andreas Thomas, Hiu Wing Cheung, Kin-Sing Wong, Terence S. M. Wan, Emmie N. M. Ho, Mario Thevis

**Affiliations:** 1 Center for Preventive Doping Research, Institute of Biochemistry, German Sport University Cologne, Cologne, Germany; 2 European Monitoring Center for Emerging Doping Agents (EuMoCEDA), Cologne, Germany; 3 Agena Bioscience GmbH, Hamburg, Germany; 4 Racing Laboratory, The Hong Kong Jockey Club, Sha Tin Racecourse, Sha Tin, Hong Kong, China

**Keywords:** Biochemical assays, Mass spectrometry, PCR-based techniques

## Abstract

Gene doping is known as the manipulation of congenital traits by gene therapeutic approaches with the intent of illicit athletic performance enhancement. A panel prototype suitable for multiplex gene doping detection by combining multiplex Polymerase Chain Reaction (PCR)-amplification with Matrix-Assisted Laser Desorption/Ionization-Time of Flight Mass Spectrometry (MALDI-TOF MS) analysis was developed and examined for its specificity and sensitivity, and its applicability in human sports drug testing programs was assessed. The panel comprises 20 assays for exon-exon-junction detection of seven human transgenes (*EPO, FST, GH1, IGF1, MSTN* (propeptide), *VEGFA, VEGFD*), which have been considered as material to routine doping controls, in one reaction. Alongside, a suitable reference material (RM) was designed and tested for its utility. An estimated LOD_95_ of 1,500 cp / mL or 30 copies (cp) per reaction of the panel and 500 cp / mL or 10 cp per reaction of the RM was determined in plasmid-spiked human whole blood samples. The specificity and applicability of the panel and the RM was further determined by testing equine plasma samples obtained from an animal that received rAAV-delivered human transgenic *EPO* as well as 111 native human doping control samples.

## Introduction

Major effort is taken to ensure that athletes can compete fair and with equal chances in a doping-free environment and to discourage illegal and even harmful doping practices^1^. Each year, the World Anti-Doping Agency (WADA) publishes an updated “Prohibited List” to keep track with new emerging doping substances and methods relevant in the context of illicit sport performance enhancement^2^. Since 2003, “gene doping” has been added to the list to counteract the potential misuse of methods derived from the, at that time, up-coming field of gene therapy^3^. Gene doping describes the use of nucleic acids or their analogues as well as gene-therapeutic techniques, such as gene editing, for the purpose of altering an athlete’s native genetic makeup for performance enhancement. Though gene doping incidences were considered as a rather unlikely doping scenario for many years^4^, an ever-faster progress in gene therapies moving forward into clinical studies and practice as well as the emergence of gene doping-relevant products on the open market have raised the concern about the actual application of illicit nucleic acid-based methods and drugs in the context of competitive sports^5–7^.

The adoption of gene doping as a banned method into the “Prohibited List” has initiated multifaceted efforts for its detection. As gene doping approaches could deploy a vast range of methods and forms of nucleic acids (e.g., transgene-delivery via viral or non-viral vectors, silencing oligonucleotide drugs, gene editing systems such as CRISPR/Cas) as well as potential target sequences, it is rather likely that complementary methods will be necessary for its detection^7–9^. Current approaches range from single-plex *real-time* quantitative polymerase chain reaction (qPCR) to whole genome sequencing (WGS) techniques (reviewed in^10–12^). qPCR testing is versatile, fast and very sensitive, but it can also be limited by the number of assays which can be combined in one reaction. Next-generation sequencing (NGS) approaches can provide comprehensive information about alterations of the athlete’s genome and transcriptome and also allow for un-biased analyses. However, NGS analyses are comparably time-consuming, demanding in technical and bioinformatic resources and skills, and are still cost-intensive. And, the ethical concerns associated with broad sequencing techniques in human prevail, potentially complicating the application of the technology for sports drug testing purposes. To date, only one gene doping test protocol, a single-plex qPCR procedure for the detection of human transgenic erythropoietin (*EPO*), has been reported as accredited by WADA^13–16^.

**Fig. 1:**
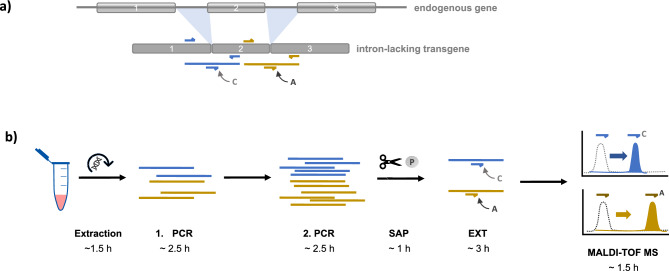
Illustration of the gene doping (GD) panel detection strategy (**a**) and process chart of the method (**b**). a) An artificial transgene can be distinguished from an endogenous gene due to the presence of intron-lacking exon-exon junctions (EEJ, exons are displayed as grey bars). The GD panel approach applies a high multiplex PCR-amplification step followed by a single base extension (SBE)-reaction over EEJ-sites. The PCR amplicons are indicated as blue and yellow bars, primers as blue and yellow small horizontal hooks. An exemplary SBE with an “A” or “C” nucleotide of an EEJ-sequence-binding extension primer is indicated with grey arrows. (**b**) Upon DNA-extraction via a spin-column based procedure, the multiplex PCR is run. The sensitivity of the method is increased by addition of a second PCR step into the method. Upon dephosphorylation and thus inactivation of excessive dNTPs via shrimp alkaline phosphatase (SAP), the SBE-reaction (EXT) is performed via addition of the extension primers and ddNTPs. The SBE-products can then be detected via MALDI-TOF MS as extended primers will lead to a mass shift in the MS-detection spectra. Approximate run times of the different steps are indicated in hours (h).

Mass Spectrometry (MS) is a broadly employed analytical technique in doping control laboratories. Liquid Chromatography Tandem Mass Spectrometry (LC–MS/MS), Quadrupole-Time-of-Flight (Q-TOF) Tandem Mass Spectrometry, and Matrix-Assisted Laser Desorption/Ionisation-Time of Flight Mass Spectrometry (MALDI-TOF MS) have successfully been tested for their applicability for the detection of chemically-modified oligonucleotides (in human and horse), however, so far have lacked sensitivity as compared to PCR-based methods^17–19^. Consequently, coupling of PCR amplification and MS as a tool to increase the detection sensitivity while taking advantage of the high multiplex detection potential of the spectrometric methods has been explored^20^. In the herein presented approach, a panel prototype has been developed for the detection of seven potential human gene doping-transgenes in one reaction. These transgenes are considered relevant for improving muscle strength as well as endurance by increasing muscle oxygenation (erythropoietin (*EPO*), vascular endothelial growth factors A and D (*VEGFA*, *VEGFD*)) or muscle mass (follistatin (*FST*), growth hormone (*GH1*), insulin-like growth factor I (*IGF1*), myostatin (propeptide, *MSTN*)) and some of them even already have entered gene therapy clinical trials^21–25^. The detection procedure exploits a single nucleotide polymorphism (SNP)-detection approach by MALDI-TOF MS originally dedicated for sample genotyping (called iPLEX^®^) and combines it with the current strategy of gene doping detection via exon-exon-junction amplification (as intron-lacking exon-exon junctions in complementary DNA (cDNA)-sequences can indicate the presence of an exogenous transgene)^12,26–28^. In the combined approach, a 20-plex PCR reaction is followed by single base-extension (SBE) of short oligonucleotide primers (called extension primers, EP) with the help of dideoxynucleotide triphosphates (ddNTPs) right over the exon-exon junction sites (Fig. 1). For an increase of sensitivity, an additional PCR-step (before the SBE-reaction) is included into the method. The single base-extended primers (SBE-products) can then be ionized by MALDI and detected by TOF–MS as the (mainly singly charged) ions can be identified according to their characteristic mass to charge ratio (*m/z*). In the presented study, the panel prototype was evaluated for its performance and suitability as a new multiplex detection method for gene doping in humans.

## Results and discussion

### Gene doping panel design

The gene doping (GD) panel was designed as a multiplex panel to test for the presence of transgenic human *EPO, FST, GH1, IGF1, MSTN, VEGFA,* and *VEGFD* cDNA in one reaction. As some of these genes are transcribed into multiple protein-coding isoforms and as it was intended that every transgene should be covered with at least two detection assays, the assay design resulted in a 19-plex detection panel plus one additional intronic assay to detect for the presence of glyceraldehyde 3-phosphate dehydrogenase (*GAPDH*) as control for successful (genomic) DNA isolation in the extraction procedure (Table 1). Supplementary Table 1 displays all (reviewed) protein-coding transcript isoforms, which can presumably be detected as cDNA with the GD panel. Though not all isoforms might be physiologically relevant in the context of sports performance enhancement, illicit use of gene doping methods still might comprise alternative variants to evade detection and were therefore considered for a comprehensive panel design^29–31^. Furthermore, whenever possible (with the exception of *MSTN*), assay designs were focused on the mature peptide sequences of the coding sequences (CDS) of the targeted genes to allow also for the detection of possibly truncated constructs. C- or N-terminally truncated constructs might show preferred characteristics like increased stability or bioactivity as well as shorter transgenic sequences could allow for better viral packing^32–35^. Particularly, since there is only one specific exon-exon junction [2()3] in the CDS of the mature peptide of *IGF1*, two detection assays using two different extension primers against the same amplicon across this specific exon-exon junction were designed (IGF1_1_1 and IGF1_1_2) to assure the testing reliability. The two additional assays of *IGF1* (IGF1_2 and IGF1_3) are for isoform-specific detection. For transgenic *MSTN*-detection, the inhibitory propeptide-sequence was considered for the assay designs^36,37^. Full assay sequences are not disclosed in order to avoid targeted genetic manipulations of transgenic sequences in a gene doping attempt. Sequences can however be shared with accredited anti-doping laboratories upon reasonable request.

**Table 1:** Overview of the 20 detection assays included in the gene doping panel. Displayed are the panel assays per target and the exon-exon-junctions which can be detected with the respective assays. An GAPDH-assay is included in the panel as genomic DNA (gDNA) control for DNA extractions.

Target	Assay	Exon()Exon Junction
*EPO*	EPO_1, EPO_2	2()3, 3()4
*GH1*	GH_1, GH_2, GH_3	2()3, 3()4, 4()5
*FST*	FST_1, FST_2	2()3, 4()5
*IGF1*	IGF1_1_1, IGF1_1_2, IGF1_2, IGF1_3	2()3, 2()3, 3()4, 3()5
*MSTN*	MSTN_1, MSTN_2	1()2, 2()3
*VEGFD*	VEGFD_1, VEGFD_2, VEGFD_3	1()2, 2()3, 3()4
*VEGFA*	VEGFA_1, VEGFA_2, VEGFA_3	2()3, 3()4, 4()5
*GAPDH*	GAPDH_1	gDNA control

### Specificity testing

All assay designs were initially pretested as single-plex assays to test for their detection specificity and before combining the assays in the final 20-plex panel. As a first specificity test, the full panel set was tested in duplicate on 160 copies (cp) of human *EPO, FST, GH1, IGF1, MSTN, VEGFA*, and *VEGFD* cDNA cloned into the pcDNA3.1( +) vector. For *GH1* and *IGF1*, two different isoforms were included into the analyses in order to cover all possible exon-exon junctions detectable with the panel. Water was analyzed as non-template controls (NTC). All assays of the panel showed a specific detection and were called only in the presence of the respective transgenic construct. Supplementary Fig. 1 displays exemplary detection mass spectra of the transgenic sequences as well as for the non-template controls. Of note, the detection specificity for *EPO* and *IGF1* was previously shown in the context of assumed gene doping product testing^6^. Detection specificity of the *GAPDH*-assay was evaluated by spiking of the iPLEX^®^ reactions with human genomic DNA (gDNA) (Supplementary Fig. 2).

### Sensitivity testing

**Fig. 2:**
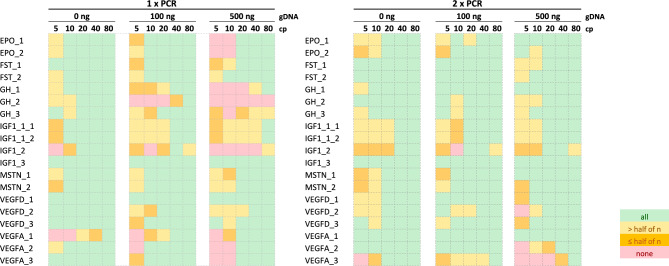
Sensitivity test of the GD panel. The panel was tested on 5 to 80 copies (cp) per reaction of transgene-containing plasmids in increasing amount of genomic DNA (gDNA) background. Analyses were run in triplicates with the one-step (1 × PCR) and two-step (2 × PCR)-PCR protocol. The assays GH_3, IGF1_1_1 and IGF1_1_2 were analyzed in 6 replicates as they are able to bind on both isoforms tested for GH1 or IGF1, respectively. The results are summarized as heatmaps. Green = all replicates, light yellow = more than half, dark yellow = half or less, red = none of the replicates were called positive.

**Fig. 3:**
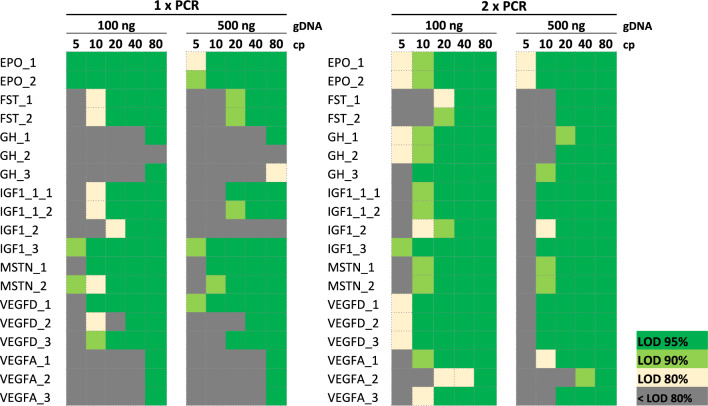
LOD results of the GD panel summarized as heatmaps. The GD panel was tested on two different serial dilutions of pooled transgenic plasmids with increasing amount of gDNA background. Each assay was tested in 12 replicates with the one-step (1 × PCR) and two-step (2 × PCR)-PCR protocol. GH_3, IGF1_1_1 and IGF1_1_2 were tested in 24 replicates as they were analyzed on two different isoforms. Green = LOD_95_, light green = LOD_90_, yellow = LOD_80_, grey = below LOD_80_.

For a first sensitivity assessment, the panel was tested in triplicates on two-fold serial dilutions of the various plasmids (from 80 to 5 cp) with increasing amounts of human gDNA background (100 ng and 500 ng, Fig. 2). A genomic background of up to 500 ng was chosen as this amount represents a likely average background scenario for DNA extractions from 1 mL of whole blood (see also section “Test of panel on spiked human whole blood samples”). Without gDNA background, most of the assays allowed for the reproducible detection of 10–20 cp per reaction. However, with increasing amounts of gDNA background, especially the detection assays for *GH1* and *IGF1* showed a significant performance drop. To increase the detection performance, the iPLEX^®^ protocol was extended by an additional PCR step, which includes a stepwise adjustment of the annealing temperature (a more detailed description of the *touch*-*down* PCR protocol can be found in the Methods section). With the two-step PCR protocol, most of the assays’ sensitivities could successfully be optimized to 20 cp even in higher amounts of gDNA background. Only the *VEGFA*-assays 2 and 3 still showed a lower detection sensitivity with 40–80 cp per reaction compared to the other assays. Attributed to the generation of a larger amplicon product, the IGF1_3 assay showed a reduced performance on the isoform NM_001111283.3. In the summarized heatmap results, the IGF1_3 assay performance was therefore only considered for the isoform for which the assay was originally developed (NM_000618.5, Supplementary Table 1). Noteworthy, the assays IGF1_1_1, IGF1_1_2 and GH_3 in general performed slightly lower on the isoforms NM_022560.4 (*GH1*) and NM_001111283.3 (*IGF1*) (which was also observed in the human whole blood spike-in study, see results (Pool B, PB) below), which might be due to differences in the secondary structure of the constructs. In order to test the assays in higher replicate numbers according to ISO20395^38^, the panel was further retested on two different serially dilutions of pooled plasmids (referred to as “Pool A” and “Pool B”; the Methods section and Supplemental Table 1 indicate the plasmid isoforms contained in the respective pools). Figure 3 summarizes the limit of detection (LOD) results for all assays as LOD_95_, LOD_90_, and LOD_80_ (Supplemental Fig. 3 shows the test performance of the assays in actual replicate numbers). Using the two-step PCR protocol, all assays were able to achieve an LOD_95_ of 20–40 cp per reaction in gDNA background, except for VEGFA_2, which showed an LOD_95_ of 80 cp per rxn. The lower sensitivity of the assay was deemed as acceptable as most of the *VEGFA*-isoforms can be detected with high sensitivity with the two other *VEGFA* assays. Noteworthy, the two *EPO*-assays were able to achieve an LOD_95_ of 20 cp and an LOD_90_ of 10 cp per reaction. Great care was required to avoid cross-contaminations during method development. Over the course of the above-mentioned experiments, erroneous assay calls were observed twice for the IGF1_3 in one NTC reaction on two different plates, and once for the EPO_2 assay in one reaction of the plasmid pool B (which does not contain the *EPO*-plasmid). These occurred with the one-step PCR protocol (1 × IGF1_3) and the two-step PCR protocol (1 × IGF1_3, 1 × EPO_2) while none were encountered during the testing of high numbers of human athlete samples in which no wildtype constructs were tested alongside (see below). The *GAPDH*-assay called positive only in gDNA-containing samples as expected.

### Design and testing of reference material

**Fig. 4:**
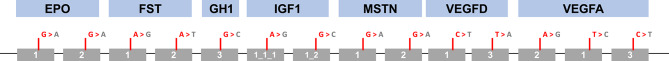
Schematic representation of the designed reference material (RM). Grey bars indicate the assay sequences represented in the RM. The red lines and letters (red: WT, grey: MUT) schematically indicate the single nucleotide mutation-sites in the RM sequence which are represented as multiple “MUT”-labeled peaks in the MassARRAY® spectrum.

**Fig. 5:**
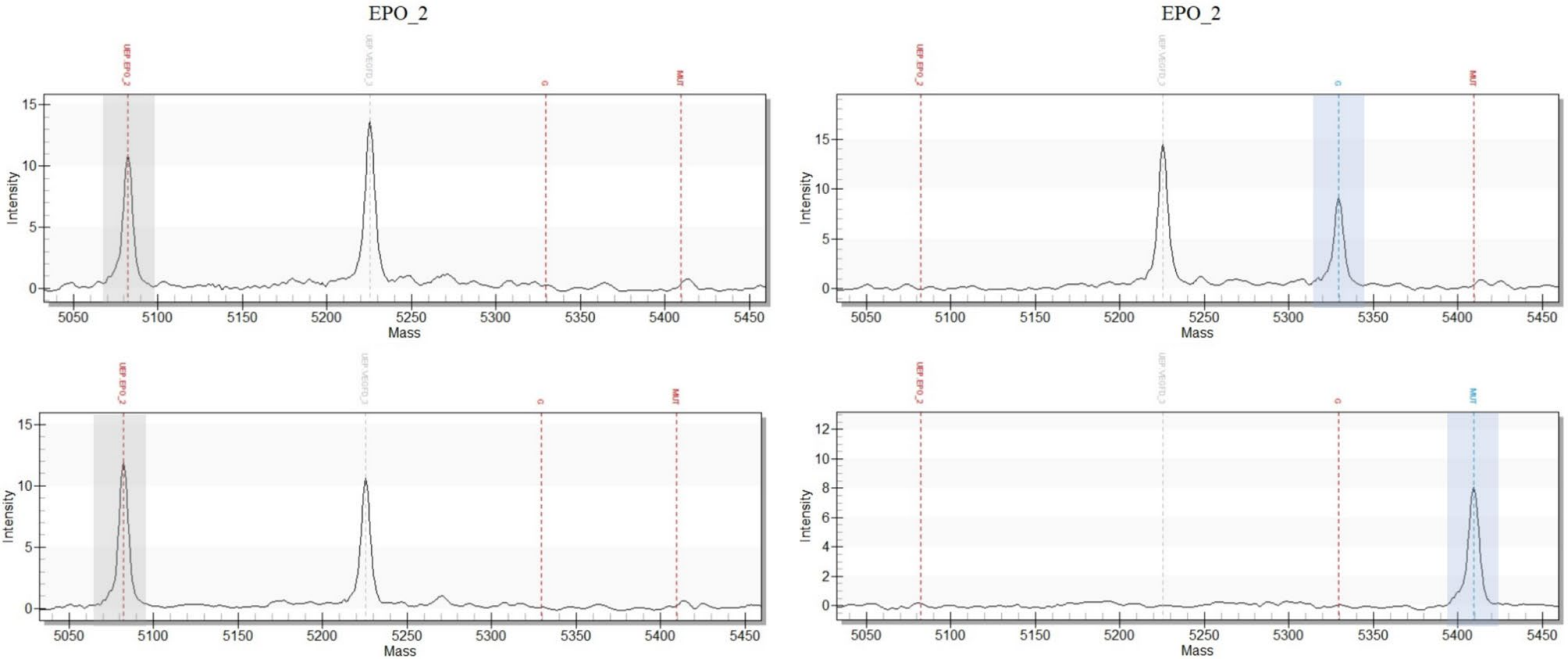
Comparative spectra for the detection of either an *EPO*-transgene containing plasmid or the RM with the EPO_2 assay of the GD panel. Left spectra show the NTCs, right spectra the detection of 80 cp of either the RM (lower spectrum) or the *EPO*-plasmid (upper spectrum). In each spectrum, red dotted lines on the left indicate expected masses of unextended extension primers (UEPs), red dotted lines on the right expected masses for the SBE-products of the wildtype sequence and of the respective mutated sequence on the RM (MUT). Called assays are indicated with a blue dotted line and were additionally highlighted with a blue bar. UEP peaks in the NTCs are highlighted with a grey bar. (The peak at the grayed-out line at 5225.4 Da originates from the UEP of the VEGFD_3 assay. As the sequence is present in the RM, the UEP peak disappears from the EPO_2 spectrum upon extension on the RM (lower right spectrum).)

**Fig. 6:**
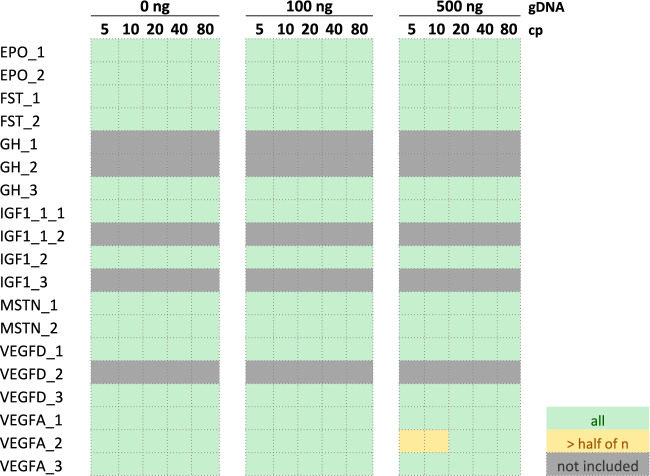
Performance testing of the RM with the GD panel. Five to 80 cp of the RM were spiked into the iPLEX^®^ reaction with increasing amounts of gDNA and tested with the two-step PCR protocol. Each input concentration was tested in 12 replicates. The assays GH_1, GH_2, IGF1_1_2, IGF1_3, VEGFD_2 and the GAPDH assay are not included on the RM and are therefore marked in grey. The results are summarized as heatmaps. Green = all replicates, light yellow = more than half of the replicates were called positive.

In contrast to qPCR analyses, the iPLEX^®^ analyses coupled to MALDI-TOF MS allows for high multiplexing but also requires a multistep workflow, which makes the method more prone to assay cross-contaminations (as seen in rare instances in the sensitivity study above). Inter-assay cross-contamination-risks are reduced by the use of deoxyuridine triphosphates (dUTPs) in the PCR mastermixes and by an uracil-N-glycosylase (UNG) digestion step preceding the first PCR-step (see the Methods section for more details on the digestion step). However, in order to avoid erroneous detections by running a wildtype (WT) construct as positive control within the method, an artificial reference material (RM) was designed, which can unequivocally be distinguished from WT sequences, and was implemented as quality control in the presented method. The RM was designed as a double-stranded, linear gene fragment (1,368 bp) comprising at least one detection assay sequence for each of the seven target genes (Fig. 4). In order to allow distinction of the RM from WT sequences, the SBE-extension sites were single point-mutated in the RM, so SBE-products of the RM (displayed as “MUT” in the mass spectra) are mass-shifted due to the incorporation of a different nucleotide compared to the WT sequence (Fig. 5). More details about the RM design can be found in the Methods section, the sequence can also be disclosed to doping control laboratories upon reasonable request. Testing of serial dilutions of the RM in gDNA background indicated that the experimental approach was feasible as the RM was detectable with very high sensitivity with the GD panel (Fig. 6; Supplementary Fig. 5 shows the same heatmap but with actual replicate numbers). Higher detection sensitivity is probably due to the lower sequence complexity and linear structure of the RM (as compared to the supercoiled WT plasmids). A circular format of the RM could decrease the detection sensitivity to levels comparable to that observed with the plasmid-inserted transgenes and could thus be considered in future investigations. Although the RM in this study was detected with superior sensitivity compared to the WT constructs, it effectively allows monitoring the successful DNA extraction and iPLEX^®^ reaction.

### Panel optimization

**Fig. 7:**
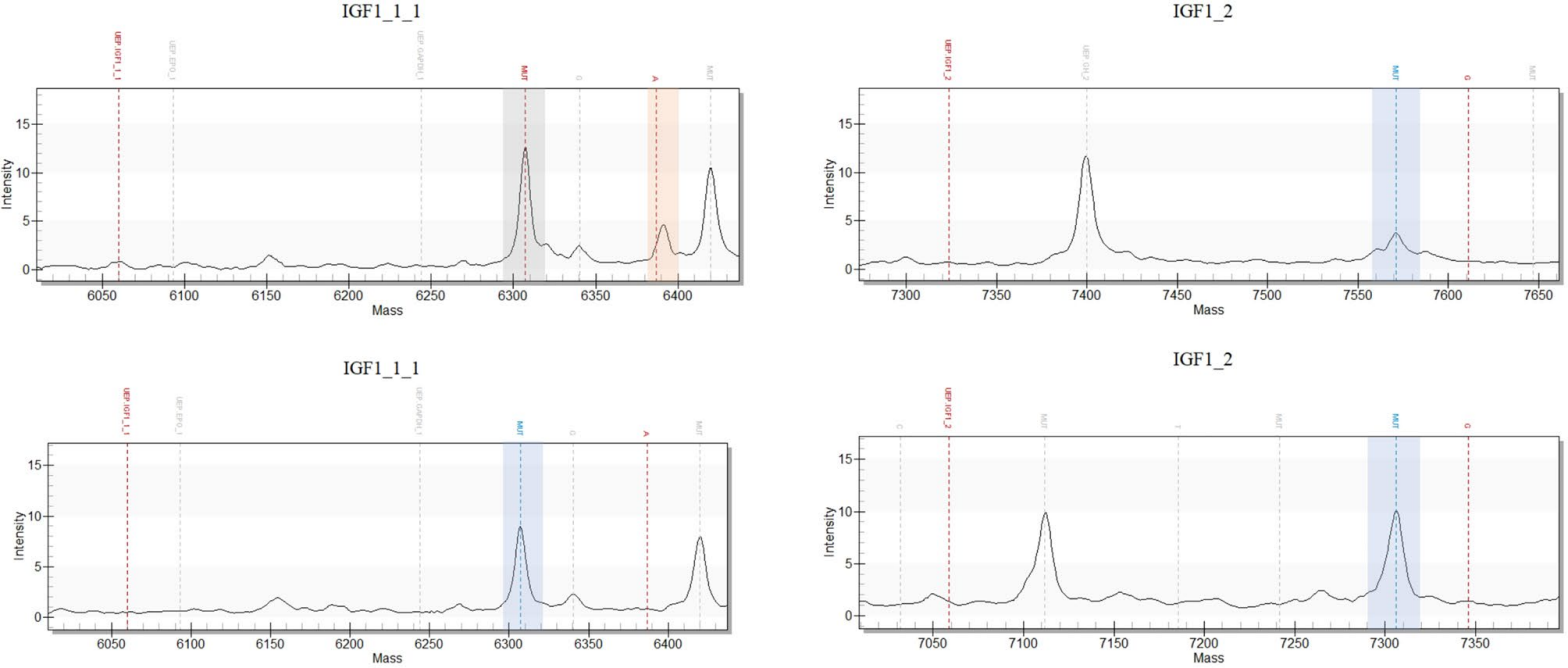
Degradation of the IGF1_2 extension primer (EP) in the panel version v1.0 and stabilization of the assay in the panel version v.1.1. Shown are exemplary spectra for the analysis of the RM (80 cp) with the panel version v1.0 (upper spectra) and v.1.1. (lower spectra). Upper spectra: Degradation of the IGF1_2 EP leads to an erroneous peak (highlighted with a red bar) in the spectrum of the IGF1_1_1 assay. IGF1_1_1 MUT calling by the TYPER software is disturbed by the unexpected EP-degradation peak (grey bar, upper left spectrum). Lower spectra: Upon PTO-stabilization of the IGF1_2 assay in the panel version v1.1, no degradation of the IGF1_2 EP and thus no erroneous peak in the IGF1_1_1 spectrum is seen anymore. Called MUT assays are highlighted with a blue line and bar. (Peaks marked with grayed-out dotted lines correspond to the MUT-extended EPO_1 assay (IGF1_1_1 spectra), the UEP of the GH_2 assay (IGF1_2 upper spectrum) and the MUT-extended VEGFD_1 assay (IGF1_2 lower spectrum)).

With testing of the RM and transgenic plasmids with the GD panel in higher replicate numbers, a more detailed picture of the assay performances was obtained. Manual correction of calls was occasionally required and only conducted when a signal-to-noise (SNR) ratio > 5.00 and a probability > 0.99 was given. The repetitive analysis over different enzyme LOTs indicated a degradation issue concerning the IGF1_2 extension primer, attributable to an increased intrinsic exonuclease activity of the PCR enzyme and, especially, within the two-step PCR approach (Supplementary Fig. 6). The degradation was recognized by the fact that the unextended extension primer (UEP) peak disappeared from the spectrum, while only a very small or no extended extension primer (EP) peak was seen for the IGF1_2 assay. As a consequence, the IGF1_2 extension primer was exchanged and stabilized with phosphorothioates (PTO, Fig. 7). Furthermore, as the GH_2 assay showed a WT extension site close to the IGF1_1_2 UEP detection site (Supplementary Fig. 1), this assay was mass-shifted also with the help of PTOs. Supplementary Fig. 7 illustrates exemplary spectra for the PTO-stabilized the GH_2 assay. The resulting optimized panel (v1.1) was retested in triplicates on the serial diluted plasmid pools as well as on the RM with and without gDNA background (Supplementary Fig. 8), yielding performances comparable to the previous panel prototype and, as intended, the IGF1_2 assay extension primer was successfully stabilized. The optimized panel was thus considered fit for purpose for further testing.

### Test of panel on spiked human whole blood samples

To evaluate the specificity and obtained LOD levels in actual human blood samples, eight human whole blood (WHB) samples were spiked with 1,500 or 500 cp per mL of the various transgenic plasmids in pools or the RM or were left as blank samples (as described in the WADA Laboratory Guideline “Gene Doping Detection based on Polymerase Chain Reaction”^16^). The DNA was extracted from 1 mL WHB with a silica membrane-based extraction procedure and the samples were eluted from the columns with 100 µL of water (for further details on the kit and extraction protocol used, the readers are referred to the Methods section). Assuming a recovery-rate of 100% of the templates and an input of 2 µL into the iPLEX^®^ reaction, an approximate input of 30 or 10 cp into the PCR reactions can be expected. In all samples, 1,500 cp per mL of the various plasmids were detectable with the respective detection assays, even the IGF1_3 assay performed well also on the *IGF1*-isoform NM_001111283.3 (Table 2). Five hundred cp per mL were also detectable in most but not all of the spiked-samples with all assays. The *EPO* and *MSTN* plasmid were detectable with both corresponding assays in all of the eight spiked samples, though not all sample extracts tested positive in duplicate. No plasmids were detectable in the blank samples confirming the specificity of the panel. The *GAPDH*-gDNA control assay called positive in all extractions as expected. The extracted samples showed on average a nucleic acid (NA) content of 185 ± 77 ng / µL and thus an average PCR reaction input of 369 ± 154 ng (highest input: 604 ng, lowest input: 166 ng). No correlation between higher DNA input amounts and assay performance was observed. The obtained results thus suggest an LOD_95_ of 1,500 cp per mL of human whole blood or approximately 30 cp per reaction for the 20-plex GD panel. As a comparison, qPCR testing for detection of human transgenic *EPO* (the only WADA-accredited test protocol for human transgenic gene doping so far) allows for a sensitive detection down to 10 cp per reaction. Despite, it is not as sensitive as the single-plex qPCR assay, the presented GD panel offers a multiplex testing option for more cost-effective monitoring of multiple transgenes. As the panel provides at least two different assays for each targeted transgene in one procedure, the risk of false-negative analyses (e.g. due to the introduction of sequence mutations) is reduced. Noteworthy, the tested vectors were not linearized before detection as it is proposed for qPCR-detection, which could have enhanced the detection performance. The corresponding assay sequences on the linear RM material were readily detectable in all samples and in all replicates down to 500 cp / mL or approximately 10 cp per reaction (Table 2).

**Table 2:** Test of spiked human whole blood samples (WHB) with the GD panel. Human WHB samples (n = 8; 4 f., 4 m; 1 mL) were spiked with 500 or 1,500 cp of the plasmid pools A (PA), B (PB) or the RM or were analyzed as blank (B) samples. DNA extracts were analyzed in duplicate with the GD panel (v1.1) with the two-step PCR protocol. Numbers indicate positive samples in relation to the overall number of samples (positive samples / overall samples). Numbers in brackets indicate the number of samples called positive in only one replicate. n.d. = not detected (sequence is not included in pool or RM), rxn = reaction.

	B	PA		PB		RM	
cp/mL	0	500	1.500	500	1.500	500	1.500
cp/rxn	0	10	30	10	30	10	30
EPO_1	0/8	8(2)/8	8/8	n.d	n.d	8/8	8/8
EPO_2	0/8	8(2)/8	8/8	n.d	n.d	8/8	8/8
FST_1	0/8	6(4)/8	8/8	n.d	n.d	8/8	8/8
FST_2	0/8	7(4)/8	8/8	n.d	n.d	8/8	8/8
GH_1	0/8	n.d	n.d	7(3)/8	8/8	n.d	n.d
GH_2	0/8	5(2)/8	8/8	n.d	n.d	n.d	n.d
GH_3	0/8	7(2)/8	8/8	5(2)/8	8/8	8/8	8/8
IGF1_1_1	0/8	8(1)/8	8/8	7(2)/8	8/8	8/8	8/8
IGF1_1_2	0/8	8(1)/8	8/8	7(2)/8	8/8	n.d	n.d
IGF1_2	0/8	n.d	n.d	7(2)/8	8/8	8/8	8/8
IGF1_3	0/8	8(1)/8	8/8	5(4)/8	8/8	n.d	n.d
MSTN_1	0/8	8(2)/8	8/8	n.d	n.d	8/8	8/8
MSTN_2	0/8	8(3)/8	8/8	n.d	n.d	8/8	8/8
VEGFD_1	0/8	7(1)/8	8/8	n.d	n.d	8/8	8/8
VEGFD_2	0/8	7(1)/8	8/8	n.d	n.d	n.d	n.d
VEGFD_3	0/8	7(1)/8	8/8	n.d	n.d	8/8	8/8
VEGFA_1	0/8	n.d	n.d	8(3)/8	8/8	8/8	8/8
VEGFA_2	0/8	n.d	n.d	7(3)/8	8/8	8/8	8/8
VEGFA_3	0/8	n.d	n.d	6(2)/8	8/8	8/8	8/8
GAPDH	8/8	8/8	8/8	8/8	8/8	8/8	8/8

### Transgenic human EPO detection in equine plasma

To evaluate the panel performance in actual post-administration in vivo samples, equine plasma samples collected from a thoroughbred gelding that underwent a human *EPO* transgene administration trial [intramuscularly transduced with 3 × 10^13^ viral genomes (vg) of an rAAV2/8-*hEPO* virus] were reassessed with the GD panel (v1.1). Results were compared against those achieved with the duplex qPCR assay dedicated for the detection of gene doping in horse^39^. Before the analysis of the in vivo samples, the performance of the panel was tested on spiked equine plasma samples (n = 8) with an approximate final PCR reaction input of 10 or 40 cp, respectively (Supplementary Table 2). All assays performed comparably well or even slightly better in the equine matrix, only the *IGF1*-assays did not call positive in all replicates even at the highest spiked cp number. Differences seen might be due to the lower gDNA background in the plasma samples (exact gDNA amounts could not be determined as the extraction protocol uses carrier RNA) or due to the different extraction method (magnetic beads vs. spin column) used for the equine plasma samples. As a note, the human *GAPDH*-assay was not called in the equine matrix as it generates a long (685 bp) product on equine genomic DNA and is thus not likely to perform well in the PCR reaction. After initial testing of the GD panel in the equine matrix, the horse in vivo samples were analyzed with different input amounts and conditions. The results are summarized in Table 3. Indeed, human transgenic *EPO* DNA was detectable for up to 14 days in equine plasma with the human GD panel with the two-step PCR protocol and detection results were thus comparable to the previously achieved qPCR testing results. The EPO_2 assay showed some extension primer (EP) degradation in the equine matrix, which had not been seen in the human background so far. The degradation was recognized by a significant reduction or complete disappearance of the UEP- and EP- peaks from the spectrum of the assay and the simultaneous formation of a degradation product detectable at 4182.7 Da. EP-degradation was again dependent on the exonuclease activity of the PCR enzyme and was less pronounced in a second PCR enzyme-LOT tested, and it was not seen with the one-step PCR protocol. With the one-step PCR protocol, transgenic *EPO*-DNA was repeatably detectable for up to 11 days after injection. Whereas, the two-step PCR protocol allows for a very sensitive detection, the one-step PCR protocol could therefore be sufficient as confirmation procedure for the detection of transgenic human *EPO*. Since it is not known which transgene might be used in a gene doping attempt, for the initial testing procedure the two-step protocol is recommended.

**Table 3:** In vivo detection of human transgenic *EPO* (rAAV2/8-*hEPO*) in equine plasma. Analysis results for the iPLEX^®^ duplicate reactions for the EPO_1 and EPO_2 assay run with the one-step (1 × PCR) and two-step (2 × PCR) PCR-protocol are shown. Different DNA extraction input amounts and PCR enzyme LOTs were tested. PRE = pre-administration, h = post-administration hours, d = post-administration days.

		PRE	24 h	3d	5d	7d	9d	11d	14d	28d	
100 µL input	EPO_1	(-/-)	(+ / +)	(+ / +)	(+ / +)	(+ / +)	(-/-)	(-/-)	(-/-)	(-/-)	2xPCR
	EPO_2	(-/-)	(+ / +)	(+ / +)	(-/-)	(-/-)	(-/-)	(-/-)	(-/-)	(-/-)	
400 µL input	EPO_1	(-/-)	(+ / +)	(+ / +)	(+ / +)	(+ / +)	(+ / +)	(+ / +)	(-/-)	(-/-)	1xPCR
	EPO_2	(-/-)	(+ / +)	(+ / +)	(+ / +)	(+ / +)	(+ / +)	(+ / +)	(-/-)	(-/-)	
400 µL input	EPO_1	(-/-)	(+ / +)	(+ / +)	(+ / +)	(+ / +)	(-/ +)	(-/ +)	(-/ +)	(-/-)	2xPCR
LOT 1	EPO_2	(-/-)	(+ / +)	(-/ +)	(+ / +)	(-/ +)	(+ / +)	(-/ +)	(-/ +)	(-/-)	
400 µL input	EPO_1	(-/-)	(+ / +)	(+ / +)	(+ / +)	(+ / +)	(+ / +)	(+ / +)	(-/-)	(-/-)	2xPCR
LOT2	EPO_2	(-/-)	(+ / +)	(+ / +)	(+ / +)	(+ / +)	(+ / +)	(-/-)	(-/-)	(-/-)	

### Routine athlete samples analysis

For applicability testing of the GD panel in routine procedures, the panel (v1.1) was furthermore tested on 111 native athlete WHB samples retrieved from routine doping controls (34 f, 77 m). The sample extracts were tested in duplicate in the iPLEX^®^ reaction, water was run as extraction control and iPLEX^®^-NTC. RM analyses were performed in parallel as extraction (1,500 cp / mL in 1 mL of a control WHB sample) and iPLEX^®^ reaction (40 cp / rxn) quality control. All samples (as well as the water extraction controls and NTCs) tested negative for the various transgenes detectable with the panel. Extracted samples yielded an average nucleic acid (NA)-content of 248 ± 104 ng / µL (ranging from 83 to 643 ng) and thus an average iPLEX^®^ reaction input of 496 ± 208 ng per reaction. We cannot exclude that with very high input DNA amount the reactions might had been impaired as the sensitivity study did not include gDNA background tests above 500 ng. However, the extraction control samples spiked with the RM showed an average NA content of 857 ± 155 ng / µL with the highest background tested being 1050 ng and still the RM was readily detectable in all reactions with all relevant assays. None of the athlete samples was tested positive for any of the transgenes, thus suggesting the adequate specificity of the panel and the method’s applicability for routine screening procedures.

## Conclusion

With the above results, the thoroughly characterized GD panel presented itself as a new sensitive and versatile testing tool suitable for qualitative multiplex detection of human gene doping. It was shown that seven human transgenes, namely *EPO, FST, GH1, IGF1, MSTN* (propeptide)*, VEGFA*, and *VEGFD*, which could potentially be misused for gene doping purposes are effectively detected with at least two different detection assays per target transgene. Although detection sensitivity might vary among different isoforms and the modes of delivery and vector vehicles used, sensitive detection of gene doping-relevant transgenes was achieved with various plasmid vectors and an rAAV-*hEPO* construct in human WHB samples and in an in vivo equine model, respectively. The new GD panel therefore has the potential to valuably add to the existing methods for the detection of gene doping in humans.

## Methods

### DNA plasmids and gene fragments

For detection analyses of transgenic DNA, the panel was tested on GenEZ ORF cDNA clones (GenScript, New Jersey, NJ, USA) of human *EPO* (NM_000799.4, Clone ID: OHu27940C), *FST* (NM_013409.3, Clone ID: OHu19061C), *GH1* (NM_000515.5, Clone ID: OHu27940C; NM_022560.4 Clone ID: OHu25687C), *IGF1* (NM_000618.5, Clone ID: OHu26817C; NM_001111283.3, Clone ID: OHu27428C), *MSTN* (NM_005259.3, Clone ID: OHu22435C), *VEGFD* (NM_004469.5, Clone ID: OHu27377C), and *VEGFA* (NM_001025366.3, Clone ID: OHu26685C) cloned into the pcDNA3.1(+) vector. For quantification and purity assessment, ORF cDNA clones were measured in five replicates with a NanoDrop One spectrophotometer at an absorbance of 230 nm, 260 nm, and 280 nm (ThermoFisher Scientific, Waltham, MA, USA).

The reference material (RM) was designed as a linear double stranded gene fragment (gBlock™ HiFi Gene Fragment, IDT, Coralville, IA, USA). The RM contains point mutated assay detection sequences for performance testing of the following GD assays: EPO_1, EPO_2, FST_1, FST_2, GH_3, IGF1_1_1, IGF1_2, MSTN_1, MSTN_2, VEGFD_1, VEGFD_3, VEGFA_1, VEGFA_2, and VEGFA_3. Assay sequences are separated by EcoR*I* restriction sites, in case the assay sequences need to be separated by restriction digestion. In silico restriction digestion analysis was performed using Geneious Prime Software (2023.0.4; Biomatters, Auckland, New Zealand) and NEBcutter (3.0; New England Biolabs, Ipswich, MA, USA) to confirm that digestion will not interfere with the assay detection sequences. Additionally, GFP-priming sites are included at the 5’ and 3’ end of the fragment to facilitate sequencing of the construct. The RM-sequence was NGS-verified and the fragment’s purity and quantity were assessed via triplicate spectrophotometric measurements.

### Panel design

The GD primer assays were generated with support of the Assay Designer Tool of the MassARRAY^®^ TYPER Software (v.5.0.4; Assay Design Suite v3.0) on the human genome assembly GRCh38.p14^40^. The *in-silico* designs were analyzed by BLAST for prediction of potential unspecific binding on human gDNA^41–43^. SBE-oligo binding sites at known SNP-sites were avoided by running the *in-silico* assay designs through the NCBI dbSNP database^44,45^. Sequence alignments and primer mappings were additionally supported by the Geneious Prime Software (2023.0.4; Biomatters, Auckland, New Zealand).

### Gene doping panel analysis

#### Materials

For the reaction set ups, the PCR Reagent Set (with dUTPs/UNG), the shrimp alkaline phosphatase (SAP) Reagent Set and the iPLEX^®^ Pro Reagent Set of Agena Bioscience (San Diego, CA, USA) were used. All cycling steps were performed in a Labcycler Basic System (SensoQuest, Göttingen, Germany). The PCR reactions and mastermixes were set up in an UV-cleaned PCR cabinet (Peqlab PCR Workstation Pro, Avantor, Radnor, PA, USA). For reaction set up of the second PCR-step in the two-step PCR protocol, a different PCR cabinet was used to decrease the risk of intra-assay cross-contaminations (UV cabinet, Thermo Fisher Scientific, Waltham, MA, USA). Before reaction set up, all surfaces and pipets were cleaned with DNA AWAY (ThermoFisher Scientific, Waltham, MA, USA). Filter tips and PCR-clean tubes were used (Eppendorf, Hamburg, Germany). Reactions were pipetted in 96-well PCR Frame Plates (Hamilton, Reno, NV, USA) sealed with adhesive PCR-Plate covers (Thermo Fisher Scientific, Waltham, MA, USA).

#### One-step PCR reaction

For the one-step PCR protocol, 0.625 µL PCR-grade water (VWR Chemicals, Radnor, PA, USA), 0.5 µL 10 × PCR buffer, 0.4 µL MgCl_2_, 0.1 µL dNTP/dUTP mix, 1.0 µL GD Panel PCR primers, 0.2 µL PCR enzyme (recombinant Taq Polymerase), and 0.125 µL UNG enzyme per reaction were mixed. As visual pipetting control, 0.05 µL per reaction of a 0.625 mg / mL stock solution of amaranth (Sigma Aldrich, Steinheim, Germany) was added to the mastermixes. Two µL of template or PCR-grade water (as non-template control, NTC) was added to 3 µL reaction mix to add up to a final reaction volume of 5 µL. One-step PCR cycling was initiated at 30 °C for 10 min for degradation of uracil-containing DNA-strands to reduce the risk of inter-assay cross-contaminations. After an initial heat activation of the DNA-polymerase at 94 °C for 2 min, the PCR cycling was run for 45 cycles at 95 °C for 30 s, 56 °C for 30 s, 72 °C for 1 min. The cycling was finished with a final elongation step at 72 °C for 5 min.

#### Two-step PCR reaction

For the two-step PCR protocol, two successive PCR reactions were run. As first step, the PCR reaction set up was performed as described for the one-step PCR protocol, but a different cycling protocol was used comprising the following steps: 30 °C for 10 min, 94 °C for 2 min, followed by 10 cycles at 95 °C for 30 s, 60 °C for 30 s, 72 °C for 1 min, and another 40 cycles at 95 °C for 30 s, 56 °C for 30 s, and 72 °C for 5 min. For the second PCR-reaction step, 0.8 µL PCR-grade water, 0.5 µL 10 × PCR buffer, 0.4 µL MgCl_2_, 0.1 µL dNTP/dUTP mix, 1.0 µL GD Panel PCR primers, and 0.2 µL PCR enzyme were added per reaction. Two µL of the reaction products of the first PCR-reaction were then added to 3 µL of the second PCR reaction mix for a final volume of 5 µL and the following cycling protocol was run: 94 °C for 2 min, 45 cycles at 95 °C for 30 s, 56 °C for 30 s, 72 °C for 1 min, and 72 °C for 5 min.

#### SAP reaction

Inactivation of dNTPs was achieved by adding 2 µL of an SAP-reaction mix to the reactions comprising 1.405 µL of PCR-grade water, 0.17 µL of SAP buffer, 0.3 µL of SAP enzyme, and 0.125 µL of a 0.626 mg / mL stock solution of bromophenol blue (Sigma Aldrich, Steinheim, Germany) as pipetting control per reaction. The dNTPs were dephosphorylated for 40 min at 37 °C and the reaction was terminated by heat-inactivation of the SAP-enzyme at 85 °C for 5 min.

#### EXT reaction

For the SBE-reaction (EXT), 0.57 µL PCR-grade water, 0.2 µL iPLEX^®^ Pro Buffer Plus, 0.2 µL iPLEX^®^ Pro Termination Mix (ddNTPs), 0.94 µL GD Panel Extend-Primer Mix, 0.04 µL iPLEX^®^ Pro Enzyme (recombinant Taq Polymerase) and, as pipetting control, 0.05 µL of a 0.64 mg / mL stock solution of xylene cyanol (Sigma Aldrich, Steinheim, Germany) adding up to a volume of 2 µL were added per reaction. The cycling was initiated at 95 °C for 30 s followed by 5 x [94 °C, 1 s followed by 50° C, 1 s] for 40 cycles and a final extension step at 72 °C for 30 s. As final step, 41 µL of PCR-grade water were added to each reaction adding up to a total volume of 50 µL per reaction.

#### MALDI-TOF MS analysis

The SBE-product reactions were processed automatically with a Chip Prep Module Dx (CPM) of a MassARRAY^®^ Dx Analyzer 4 (MA4) system (Agena Bioscience, San Diego, CA, USA). The reactions were desalted with 13 µL of MassARRAY^®^ Clean Resin and were then dispensed on a SpectroCHIP^®^ CPM-96 Array (dispense condition: 1200 (nL), autotune mode) using 3-hydroxypicolinic acid as crystallizing matrix. Ionization was achieved with help of the UV laser (wavelength = 337 nm) of the MassARRAY^®^ System applying a maximum number of 30 laser shots aiming at the acquisition of 5 (minimum) to 9 (maximum) spectra of acceptable quality. Raw data analysis was supported by the MassARRAY^®^ TYPER Software (v5.0.10) and by generation of genotype area reports.

### Sensitivity analyses

Plasmids were two-fold serial diluted to a final concentration of 80-40-20-10-5 cp / µL in DNA-low binding tubes (Eppendorf, Hamburg, Germany) in a background of 2 ng / µL salmon sperm DNA (Invitrogen, Carlsbad, CA, USA). For human gDNA background, pooled gDNA isolated from WHB with the QIAamp^®^ DNA Blood Midi kit (Qiagen, Hilden, Germany), see extraction procedure below, or pooled human gDNA from buffy coats was used (Roche, Basel, Switzerland). The gDNA was concentrated with help of the Genomic DNA Clean & Concentrator-25 Kit (ZYMO Research, Orange, CA, USA) according to the manufacturer’s instructions and adjusted with PCR-grade water to a final concentration of 100 or 500 ng / µL, respectively. One µL of the respective plasmid stock and 1 µL of the 100 or 500 ng / µL of gDNA were used as input into the GD panel analyses as indicated. Single-plasmid analyses were run in triplicates, pooled-plasmid analyses were run in two times six replicates of two different serial dilutions tested at different times points (n = 12). The pools tested contained the following isoforms: Pool A—NM_000799.4 (*EPO*), NM_013409.3 (*FST*), NM_000515.5 (*GH1*), NM_000618.5 (*IGF1*), NM_005259.3 (*MSTN*) NM_004469.5 (*VEGFD*); Pool B—NM_022560.4 (*GH1*), NM_001111283.3 (*IGF1*), NM_001025366.3 (*VEGFA*). Water controls were run as NTCs. The limit of detection (LOD) was defined as the lowest cp number where at least 95%, 90%, or 80% of the spiked reactions were tested positive.

### DNA extractions from human whole blood

DNA from human WHB was extracted with help of the QIAamp^®^ DNA Blood Midi kit (Qiagen, Hilden, Germany) via the following procedure: briefly, 1 mL of a WHB sample was mixed with 100 µL protease in a 15 mL tube (Falcon™, ThermoFisher Scientific, Waltham, MA, USA). After addition of 1.2 mL buffer AL, the sample was mixed again and heated for 10 min at 70 °C (Dry Block Heater 2, IKA, Staufen, Germany). Subsequently, 1 mL of 100% ethanol (Merck, Darmstadt, Germany) was added, the sample was mixed and transferred onto a QIAmp^®^ Midi column. The column was centrifuged at 1,850 × g for 3 min (Microstar 21, VWR, Rednor, PA, USA) and the filtrate was discarded. The column was washed with 2 ml buffer AW1 and afterwards with 2 mL buffer AW2 with centrifugation at 4,300 × g for 1 and 15 min, respectively. As final step, the column was placed into a new tube and the DNA was double-eluted by applying 100 µL of PCR-grade water onto the column followed by a 5 min incubation-step and centrifugation at 4,300 × *g*. The eluate was re-applied onto the column and incubated again for 5 min and was then again eluted by centrifugation for 4,300 × *g* for 5 min. The eluates were stored at -20 °C until further analysis. Two µL of the eluates were used as input for the PCR reactions which were run in duplicate per sample extract. In the WHB-spike-in study, the LOD_95_ was defined as the lowest cp number where at least 95% of the spiked samples were tested positive in both replicates. NA content and quality was assessed via duplicate spectrophotometric measurements on a NanoDrop One system (ThermoFisher Scientific, Waltham, MA, USA).

### Equine plasma sample analyses

Plasma samples were provided from a previously performed horse administration trial (for further details please refer to Cheung et al.^39^; Animal Research Ethics Committee Approval of the Hong Kong Jockey Club: ERC/035/2020). DNA extraction from equine plasma samples was done with support of the EZ1&2 Virus Mini Kit v2.0 (Qiagen, Hilden, Germany) on an EZ2 Connect Fx System (Qiagen, Hilden, Germany) according to the manufacturer’s instructions. Briefly, up to 400 µL of equine plasma was mixed with 3 µg of carrier RNA (polyA) and loaded onto the Cartridge Rack of the EZ2 System. For the isolation, the “Virus Mini Kit v2.0” program was chosen with a volume of RNAse-free elution buffer of 60 µL. The eluates were stored at -20 °C until further analysis. For the GD panel analyses, 2 µL of the DNA extracts were used as PCR input, analyses were run in duplicate with the one-step or two-step PCR protocol. For the spike-in analyses, 100 µL of equine plasma of eight different horses collected for routine analytical procedures, were spiked with 3,000 or 12,000 cp per mL of the plasmid pools A or B or 12,000 cp per mL the RM or were left as blanks.

### Participants

Human WHB samples were collected from healthy adult male and female volunteers after information of the participants. Informed consent was obtained from all subjects. The approval was given by the local ethics committee of the German Sport University Cologne (#2021/139) and research was conducted in accordance with the Declaration of Helsinki. All methods were performed in accordance with the relevant guidelines and regulations within the manuscript.

## Supplementary Information

Supplementary Information.

## Data Availability

The datasets analyzed during the current study are available from the corresponding author on reasonable request.
