# Supplementary material for: Multiplex detection of seven transgenes for human gene doping analysis
Source: Sci Rep. 2025 Jun 20;15:20219. doi: 10.1038/s41598-025-06677-4 (PMC12181312; doi:10.1038/s41598-025-06677-4)
Supplement: Supplementary file 1 — Supplementary Information. [file 41598_2025_6677_MOESM1_ESM.pptx]

## Slide 1
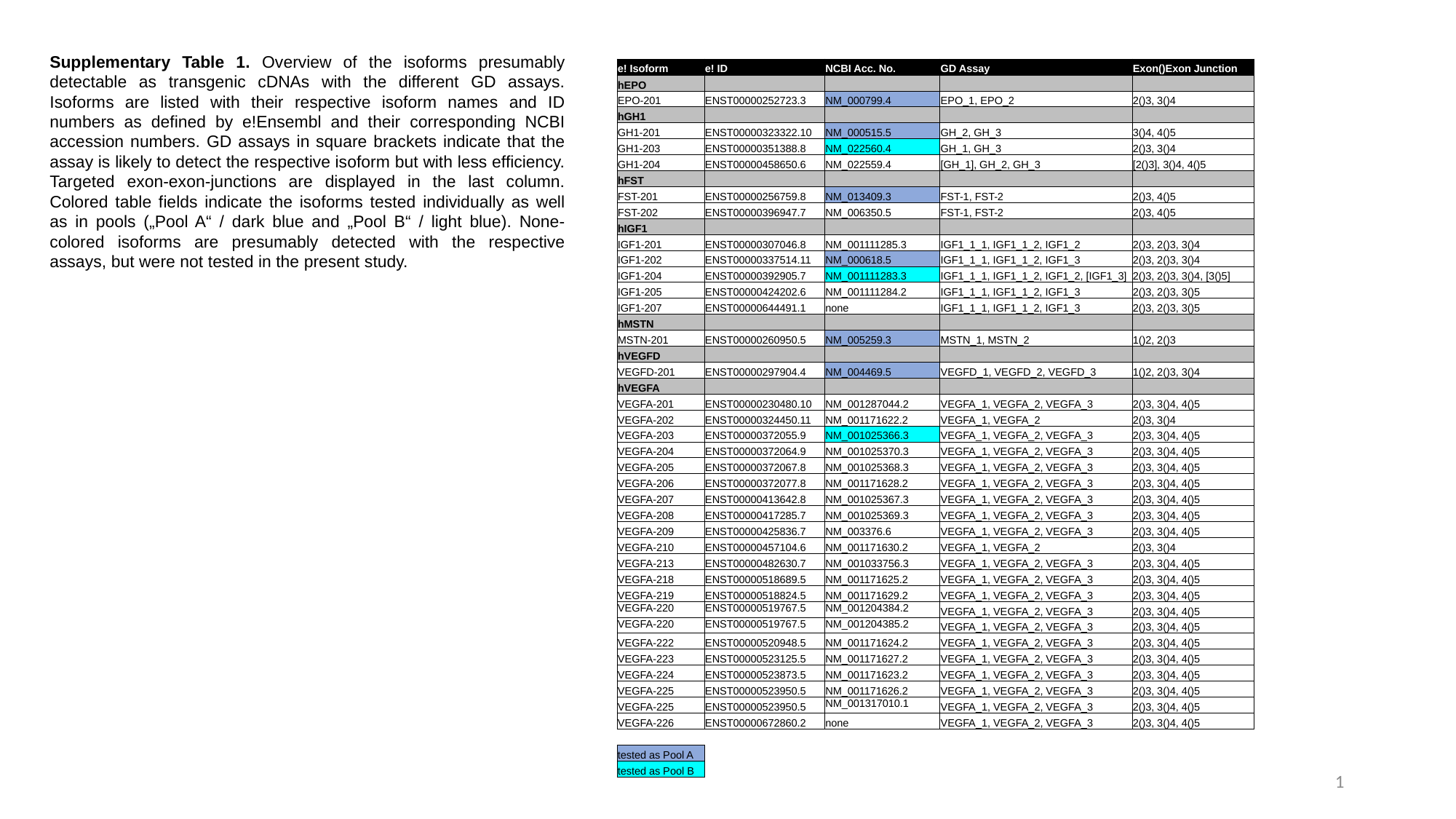

Supplementary Table 1. Overview of the isoforms presumably detectable as transgenic cDNAs with the different GD assays. Isoforms are listed with their respective isoform names and ID numbers as defined by e!Ensembl and their corresponding NCBI accession numbers. GD assays in square brackets indicate that the assay is likely to detect the respective isoform but with less efficiency. Targeted exon-exon-junctions are displayed in the last column. Colored table fields indicate the isoforms tested individually as well as in pools („Pool A“ / dark blue and „Pool B“ / light blue). None-colored isoforms are presumably detected with the respective assays, but were not tested in the present study.
| e! Isoform | e! ID | NCBI Acc. No. | GD Assay | Exon()Exon Junction |
| --- | --- | --- | --- | --- |
| hEPO | | | | |
| EPO-201 | ENST00000252723.3 | NM\_000799.4 | EPO\_1, EPO\_2 | 2()3, 3()4 |
| hGH1 | | | | |
| GH1-201 | ENST00000323322.10 | NM\_000515.5 | GH\_2, GH\_3 | 3()4, 4()5 |
| GH1-203 | ENST00000351388.8 | NM\_022560.4 | GH\_1, GH\_3 | 2()3, 3()4 |
| GH1-204 | ENST00000458650.6 | NM\_022559.4 | [GH\_1], GH\_2, GH\_3 | [2()3], 3()4, 4()5 |
| hFST | | | | |
| FST-201 | ENST00000256759.8 | NM\_013409.3 | FST-1, FST-2 | 2()3, 4()5 |
| FST-202 | ENST00000396947.7 | NM\_006350.5 | FST-1, FST-2 | 2()3, 4()5 |
| hIGF1 | | | | |
| IGF1-201 | ENST00000307046.8 | NM\_001111285.3 | IGF1\_1\_1, IGF1\_1\_2, IGF1\_2 | 2()3, 2()3, 3()4 |
| IGF1-202 | ENST00000337514.11 | NM\_000618.5 | IGF1\_1\_1, IGF1\_1\_2, IGF1\_3 | 2()3, 2()3, 3()4 |
| IGF1-204 | ENST00000392905.7 | NM\_001111283.3 | IGF1\_1\_1, IGF1\_1\_2, IGF1\_2, [IGF1\_3] | 2()3, 2()3, 3()4, [3()5] |
| IGF1-205 | ENST00000424202.6 | NM\_001111284.2 | IGF1\_1\_1, IGF1\_1\_2, IGF1\_3 | 2()3, 2()3, 3()5 |
| IGF1-207 | ENST00000644491.1 | none | IGF1\_1\_1, IGF1\_1\_2, IGF1\_3 | 2()3, 2()3, 3()5 |
| hMSTN | | | | |
| MSTN-201 | ENST00000260950.5 | NM\_005259.3 | MSTN\_1, MSTN\_2 | 1()2, 2()3 |
| hVEGFD | | | | |
| VEGFD-201 | ENST00000297904.4 | NM\_004469.5 | VEGFD\_1, VEGFD\_2, VEGFD\_3 | 1()2, 2()3, 3()4 |
| hVEGFA | | | | |
| VEGFA-201 | ENST00000230480.10 | NM\_001287044.2 | VEGFA\_1, VEGFA\_2, VEGFA\_3 | 2()3, 3()4, 4()5 |
| VEGFA-202 | ENST00000324450.11 | NM\_001171622.2 | VEGFA\_1, VEGFA\_2 | 2()3, 3()4 |
| VEGFA-203 | ENST00000372055.9 | NM\_001025366.3 | VEGFA\_1, VEGFA\_2, VEGFA\_3 | 2()3, 3()4, 4()5 |
| VEGFA-204 | ENST00000372064.9 | NM\_001025370.3 | VEGFA\_1, VEGFA\_2, VEGFA\_3 | 2()3, 3()4, 4()5 |
| VEGFA-205 | ENST00000372067.8 | NM\_001025368.3 | VEGFA\_1, VEGFA\_2, VEGFA\_3 | 2()3, 3()4, 4()5 |
| VEGFA-206 | ENST00000372077.8 | NM\_001171628.2 | VEGFA\_1, VEGFA\_2, VEGFA\_3 | 2()3, 3()4, 4()5 |
| VEGFA-207 | ENST00000413642.8 | NM\_001025367.3 | VEGFA\_1, VEGFA\_2, VEGFA\_3 | 2()3, 3()4, 4()5 |
| VEGFA-208 | ENST00000417285.7 | NM\_001025369.3 | VEGFA\_1, VEGFA\_2, VEGFA\_3 | 2()3, 3()4, 4()5 |
| VEGFA-209 | ENST00000425836.7 | NM\_003376.6 | VEGFA\_1, VEGFA\_2, VEGFA\_3 | 2()3, 3()4, 4()5 |
| VEGFA-210 | ENST00000457104.6 | NM\_001171630.2 | VEGFA\_1, VEGFA\_2 | 2()3, 3()4 |
| VEGFA-213 | ENST00000482630.7 | NM\_001033756.3 | VEGFA\_1, VEGFA\_2, VEGFA\_3 | 2()3, 3()4, 4()5 |
| VEGFA-218 | ENST00000518689.5 | NM\_001171625.2 | VEGFA\_1, VEGFA\_2, VEGFA\_3 | 2()3, 3()4, 4()5 |
| VEGFA-219 | ENST00000518824.5 | NM\_001171629.2 | VEGFA\_1, VEGFA\_2, VEGFA\_3 | 2()3, 3()4, 4()5 |
| VEGFA-220 | ENST00000519767.5 | NM\_001204384.2 | VEGFA\_1, VEGFA\_2, VEGFA\_3 | 2()3, 3()4, 4()5 |
| VEGFA-220 | ENST00000519767.5 | NM\_001204385.2 | VEGFA\_1, VEGFA\_2, VEGFA\_3 | 2()3, 3()4, 4()5 |
| VEGFA-222 | ENST00000520948.5 | NM\_001171624.2 | VEGFA\_1, VEGFA\_2, VEGFA\_3 | 2()3, 3()4, 4()5 |
| VEGFA-223 | ENST00000523125.5 | NM\_001171627.2 | VEGFA\_1, VEGFA\_2, VEGFA\_3 | 2()3, 3()4, 4()5 |
| VEGFA-224 | ENST00000523873.5 | NM\_001171623.2 | VEGFA\_1, VEGFA\_2, VEGFA\_3 | 2()3, 3()4, 4()5 |
| VEGFA-225 | ENST00000523950.5 | NM\_001171626.2 | VEGFA\_1, VEGFA\_2, VEGFA\_3 | 2()3, 3()4, 4()5 |
| VEGFA-225 | ENST00000523950.5 | NM\_001317010.1 | VEGFA\_1, VEGFA\_2, VEGFA\_3 | 2()3, 3()4, 4()5 |
| VEGFA-226 | ENST00000672860.2 | none | VEGFA\_1, VEGFA\_2, VEGFA\_3 | 2()3, 3()4, 4()5 |
| | | | | |
| tested as Pool A | | | | |
| tested as Pool B | | | | |
1

## Slide 2
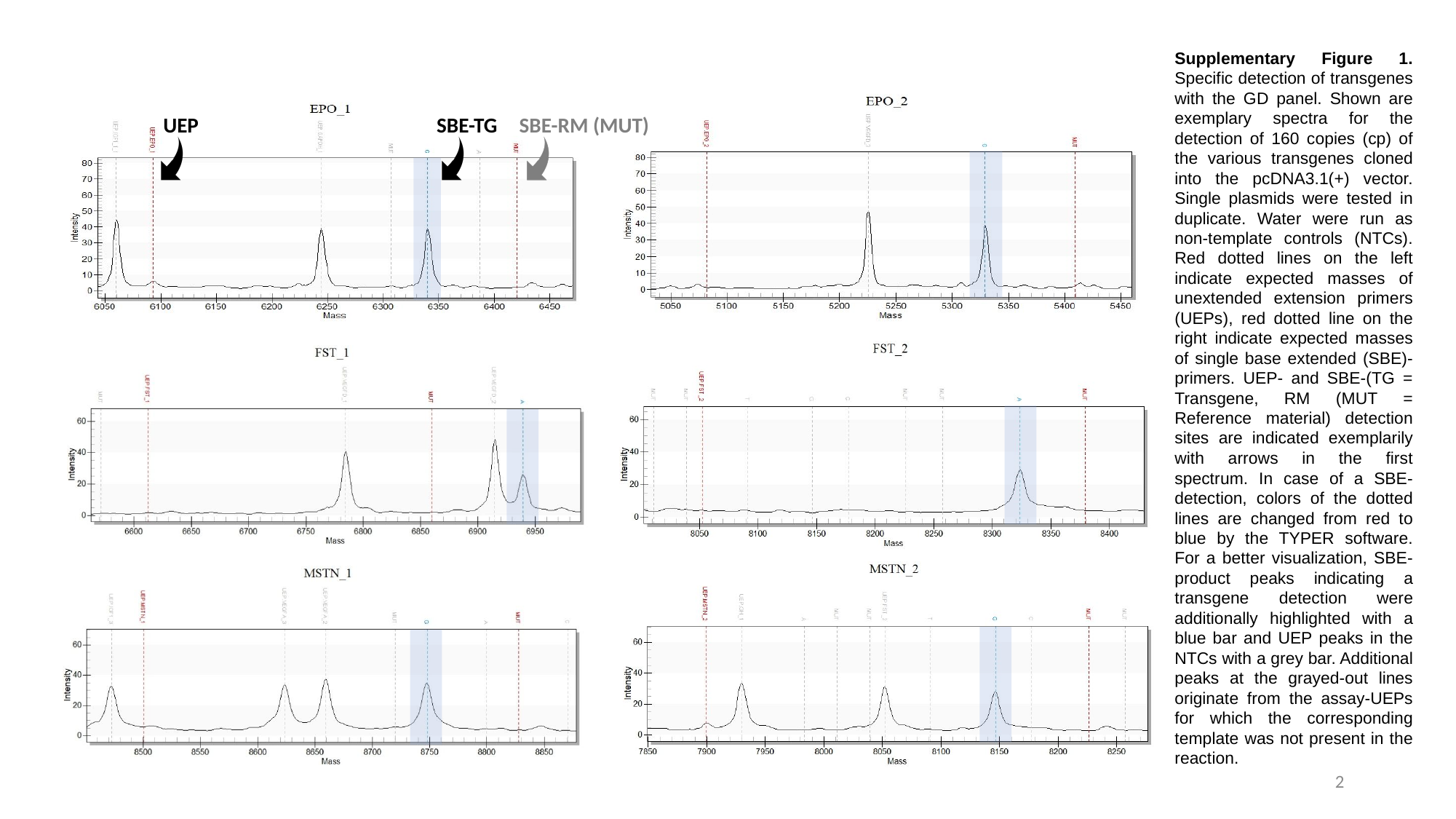

Supplementary Figure 1. Specific detection of transgenes with the GD panel. Shown are exemplary spectra for the detection of 160 copies (cp) of the various transgenes cloned into the pcDNA3.1(+) vector. Single plasmids were tested in duplicate. Water were run as non-template controls (NTCs). Red dotted lines on the left indicate expected masses of unextended extension primers (UEPs), red dotted line on the right indicate expected masses of single base extended (SBE)-primers. UEP- and SBE-(TG = Transgene, RM (MUT = Reference material) detection sites are indicated exemplarily with arrows in the first spectrum. In case of a SBE-detection, colors of the dotted lines are changed from red to blue by the TYPER software. For a better visualization, SBE-product peaks indicating a transgene detection were additionally highlighted with a blue bar and UEP peaks in the NTCs with a grey bar. Additional peaks at the grayed-out lines originate from the assay-UEPs for which the corresponding template was not present in the reaction.
UEP
SBE-TG
SBE-RM (MUT)
2

## Slide 3
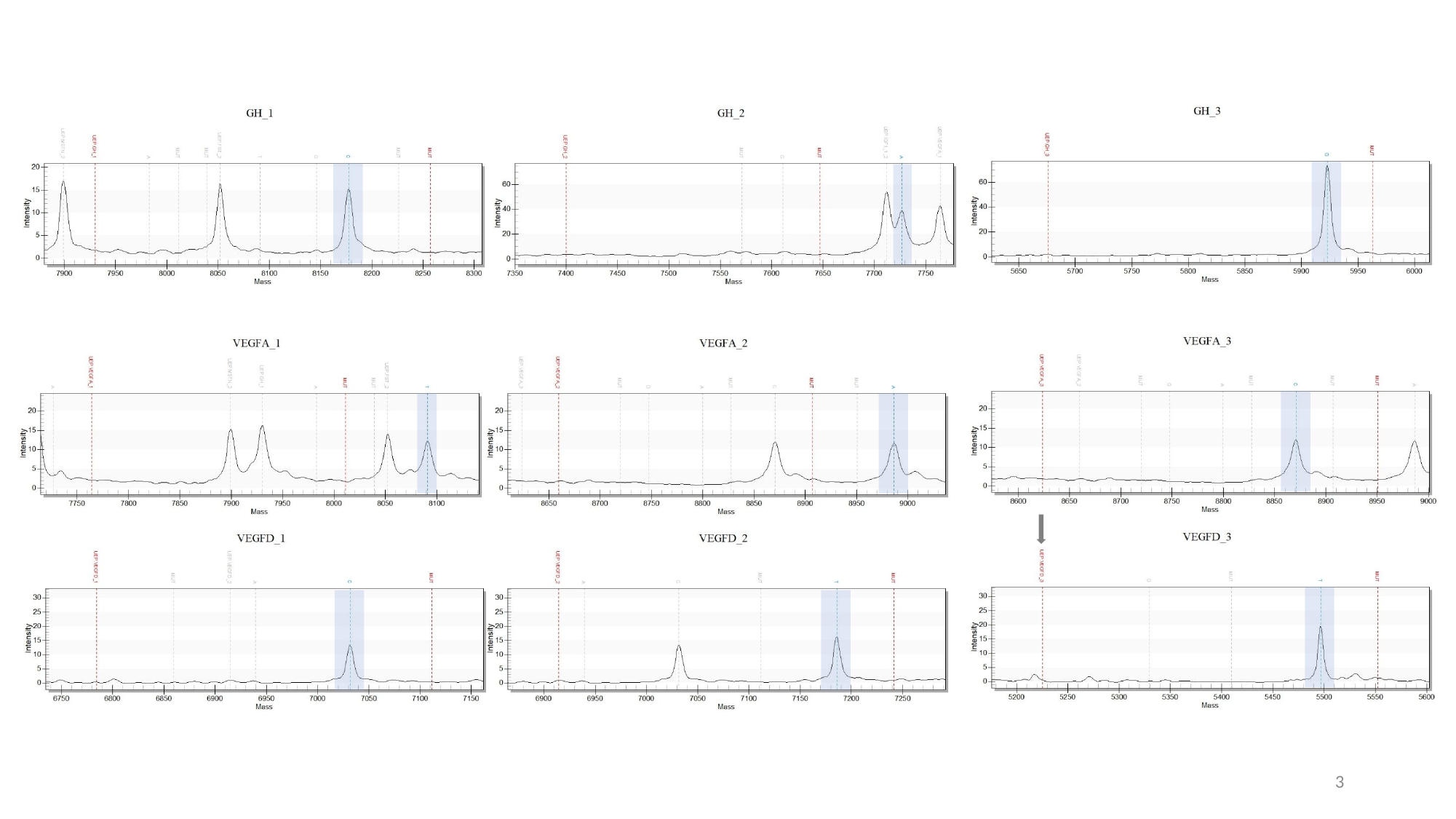

3

## Slide 4
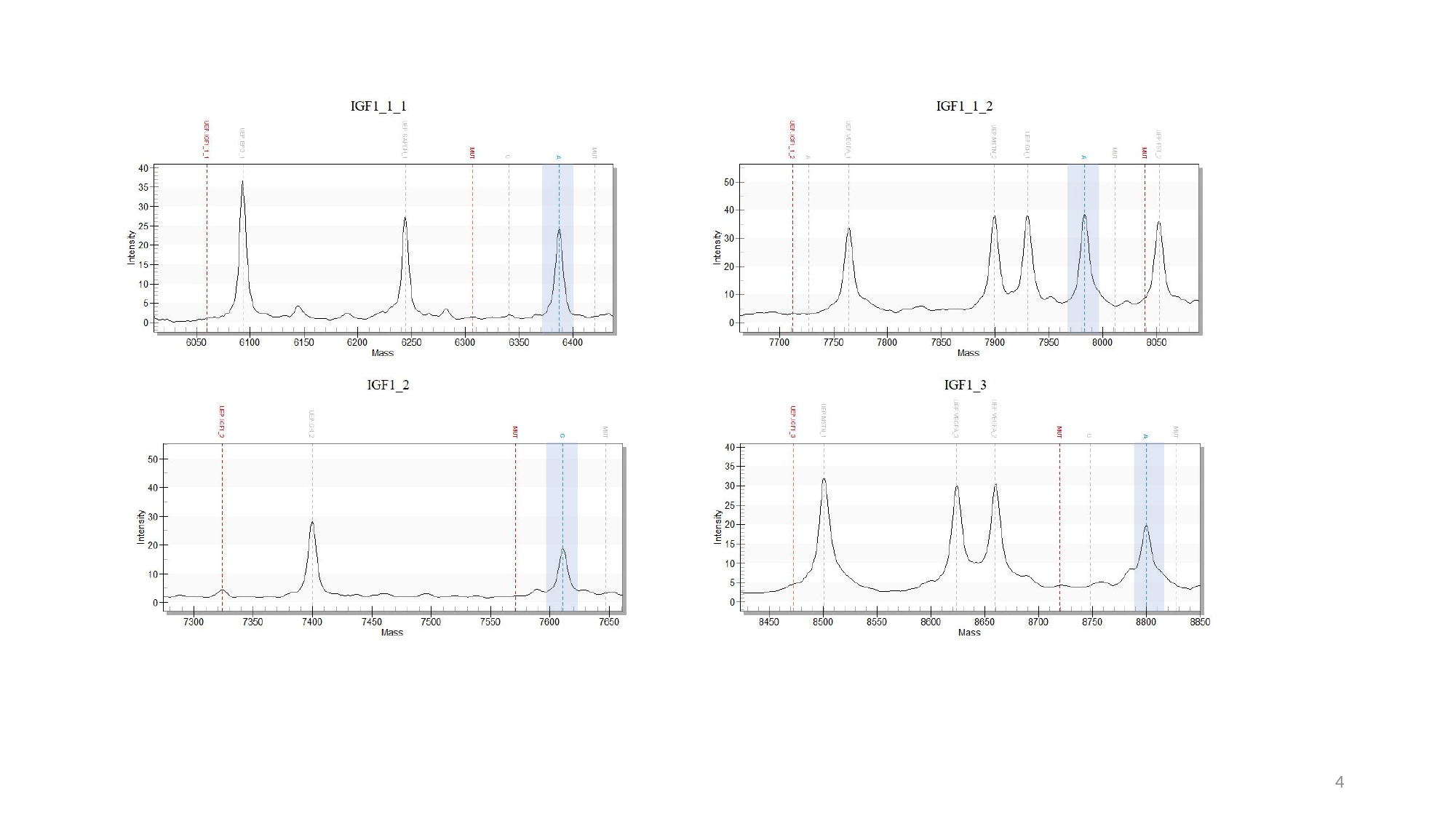

4

## Slide 5
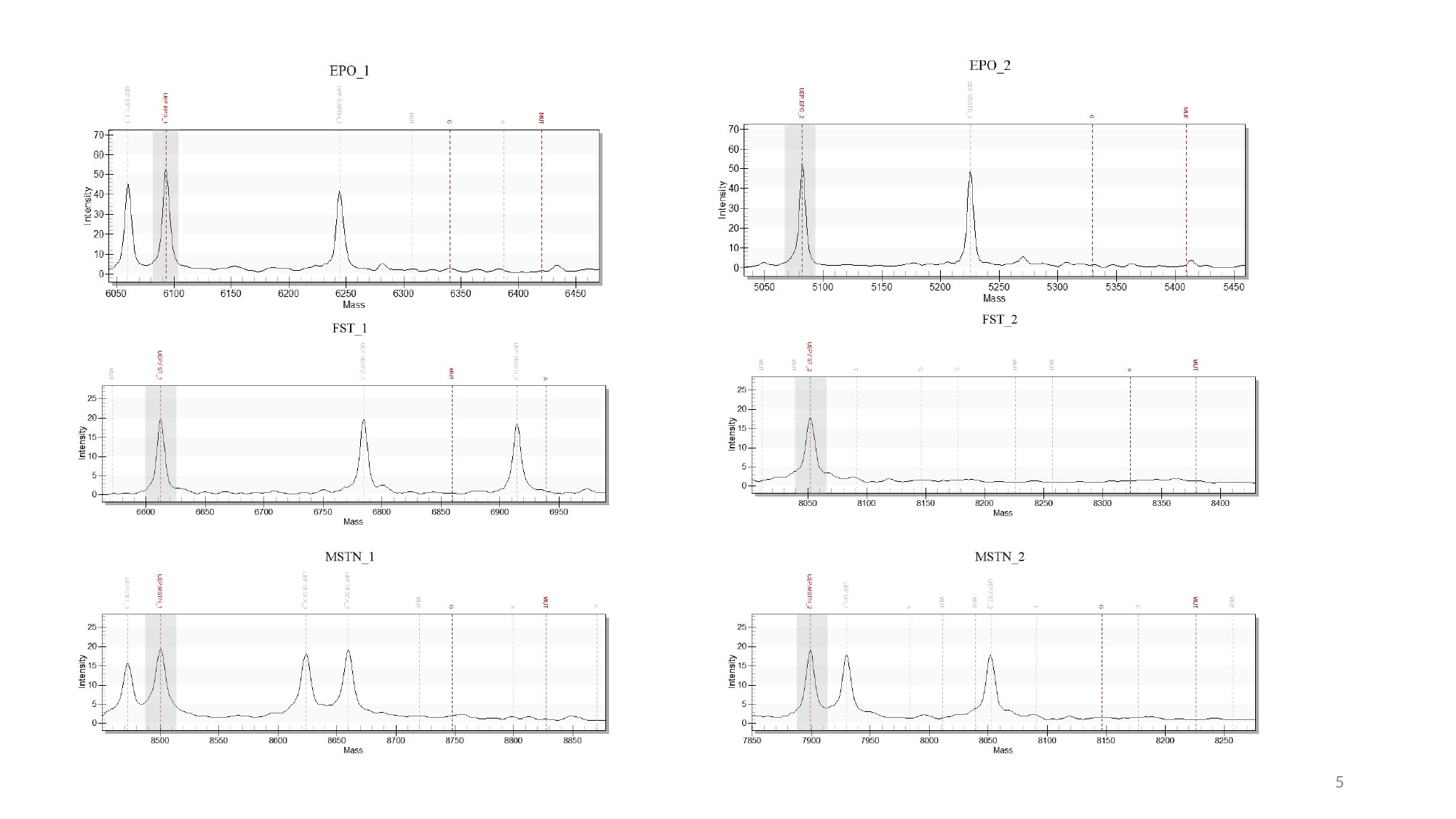

5

## Slide 6
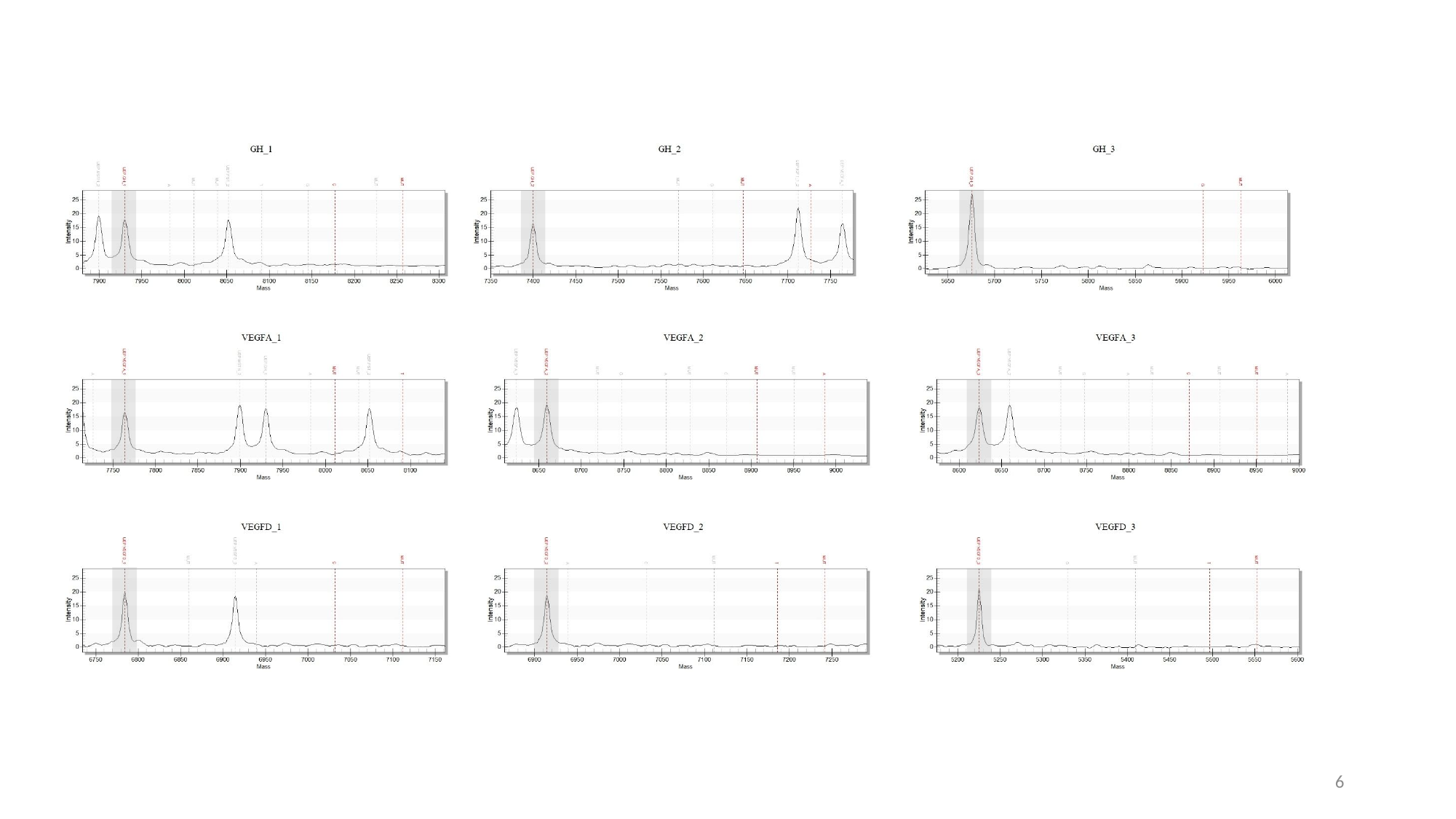

6

## Slide 7
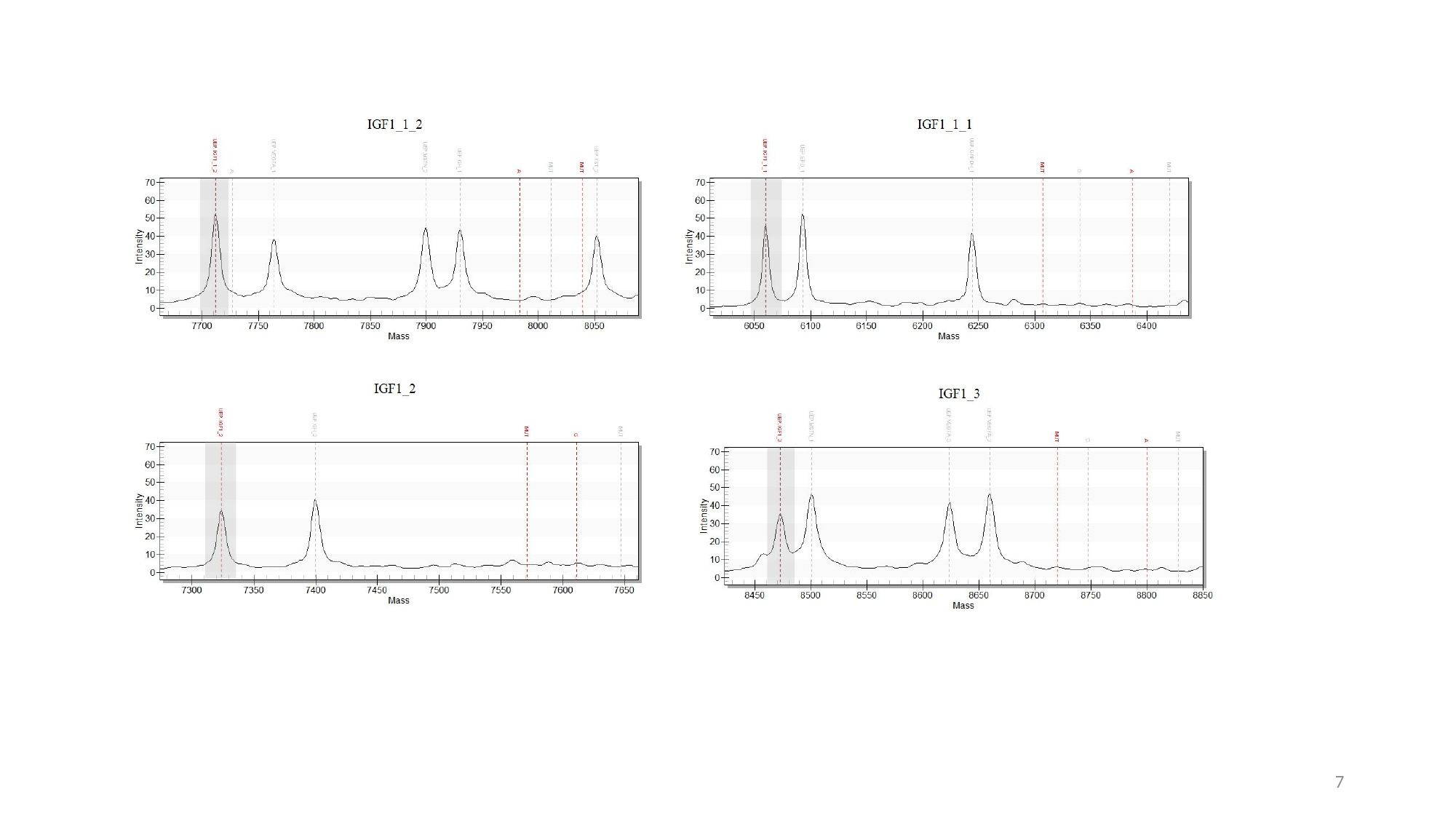

7

## Slide 8
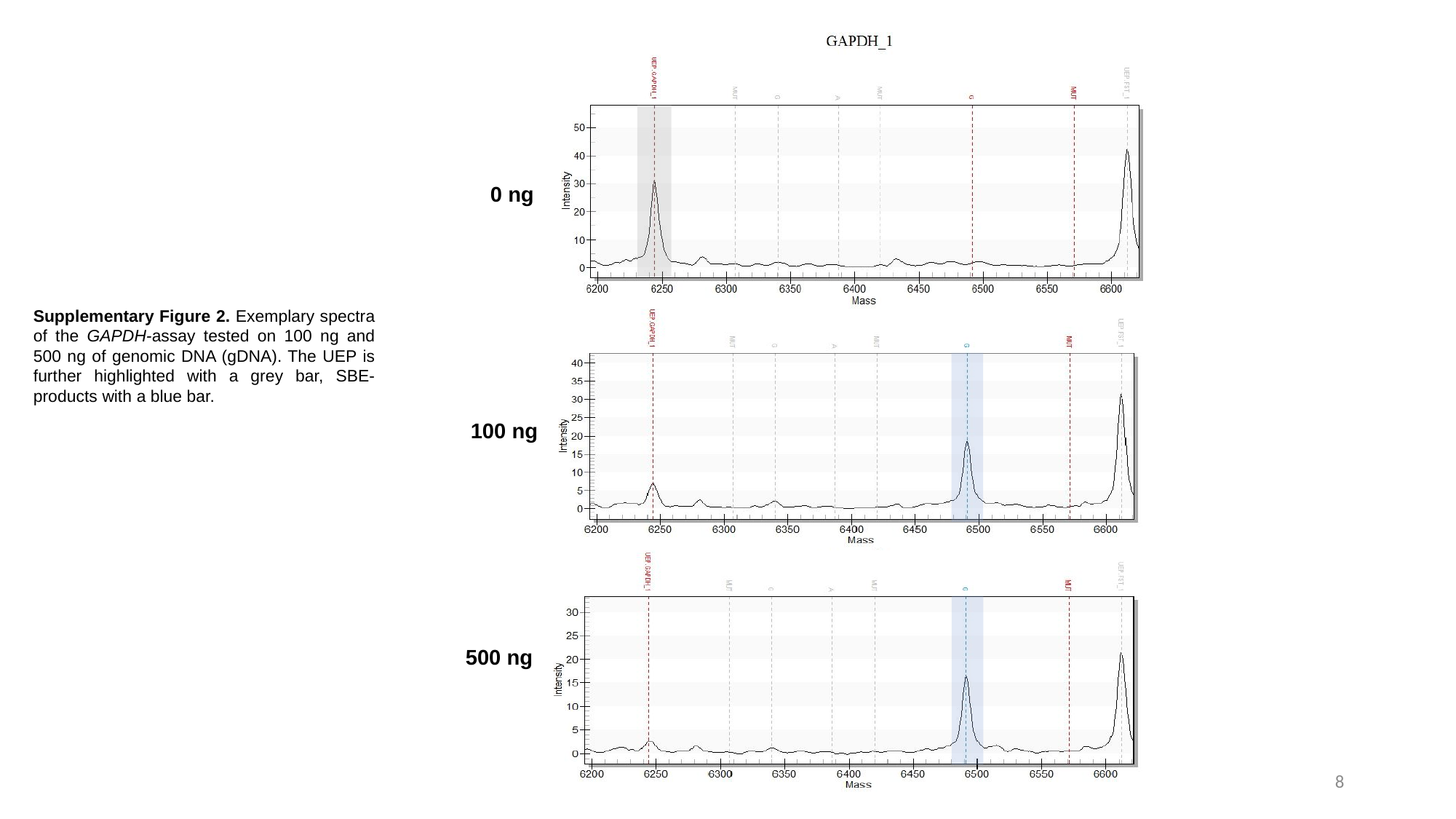

0 ng
Supplementary Figure 2. Exemplary spectra of the GAPDH-assay tested on 100 ng and 500 ng of genomic DNA (gDNA). The UEP is further highlighted with a grey bar, SBE-products with a blue bar.
100 ng
500 ng
8

## Slide 9
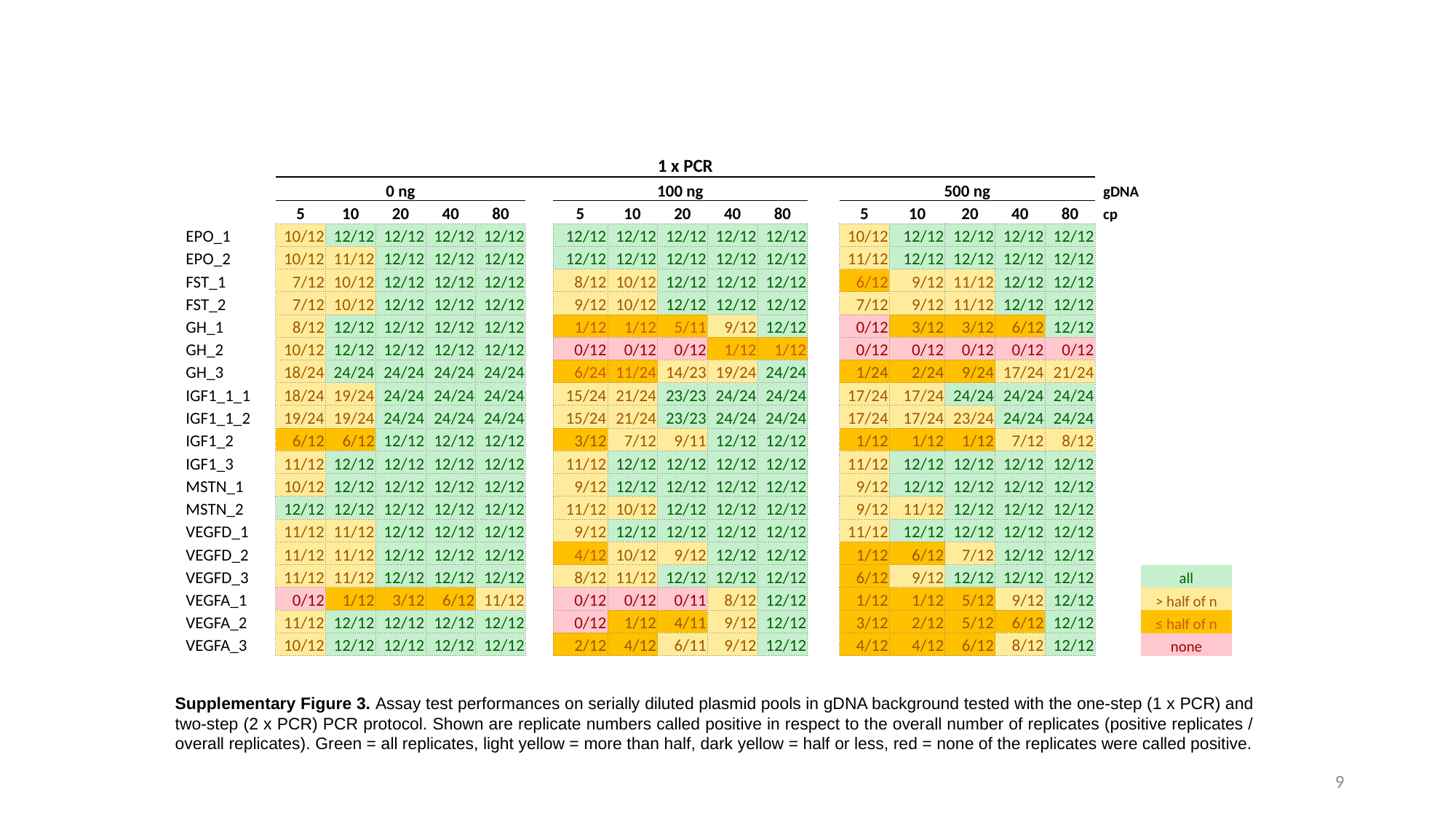

| | 1 x PCR | | | | | | | | | | | | | | | | | |
| --- | --- | --- | --- | --- | --- | --- | --- | --- | --- | --- | --- | --- | --- | --- | --- | --- | --- | --- |
| | 0 ng | | | | | | 100 ng | | | | | | 500 ng | | | | | gDNA |
| | 5 | 10 | 20 | 40 | 80 | | 5 | 10 | 20 | 40 | 80 | | 5 | 10 | 20 | 40 | 80 | cp |
| EPO\_1 | 10/12 | 12/12 | 12/12 | 12/12 | 12/12 | | 12/12 | 12/12 | 12/12 | 12/12 | 12/12 | | 10/12 | 12/12 | 12/12 | 12/12 | 12/12 | |
| EPO\_2 | 10/12 | 11/12 | 12/12 | 12/12 | 12/12 | | 12/12 | 12/12 | 12/12 | 12/12 | 12/12 | | 11/12 | 12/12 | 12/12 | 12/12 | 12/12 | |
| FST\_1 | 7/12 | 10/12 | 12/12 | 12/12 | 12/12 | | 8/12 | 10/12 | 12/12 | 12/12 | 12/12 | | 6/12 | 9/12 | 11/12 | 12/12 | 12/12 | |
| FST\_2 | 7/12 | 10/12 | 12/12 | 12/12 | 12/12 | | 9/12 | 10/12 | 12/12 | 12/12 | 12/12 | | 7/12 | 9/12 | 11/12 | 12/12 | 12/12 | |
| GH\_1 | 8/12 | 12/12 | 12/12 | 12/12 | 12/12 | | 1/12 | 1/12 | 5/11 | 9/12 | 12/12 | | 0/12 | 3/12 | 3/12 | 6/12 | 12/12 | |
| GH\_2 | 10/12 | 12/12 | 12/12 | 12/12 | 12/12 | | 0/12 | 0/12 | 0/12 | 1/12 | 1/12 | | 0/12 | 0/12 | 0/12 | 0/12 | 0/12 | |
| GH\_3 | 18/24 | 24/24 | 24/24 | 24/24 | 24/24 | | 6/24 | 11/24 | 14/23 | 19/24 | 24/24 | | 1/24 | 2/24 | 9/24 | 17/24 | 21/24 | |
| IGF1\_1\_1 | 18/24 | 19/24 | 24/24 | 24/24 | 24/24 | | 15/24 | 21/24 | 23/23 | 24/24 | 24/24 | | 17/24 | 17/24 | 24/24 | 24/24 | 24/24 | |
| IGF1\_1\_2 | 19/24 | 19/24 | 24/24 | 24/24 | 24/24 | | 15/24 | 21/24 | 23/23 | 24/24 | 24/24 | | 17/24 | 17/24 | 23/24 | 24/24 | 24/24 | |
| IGF1\_2 | 6/12 | 6/12 | 12/12 | 12/12 | 12/12 | | 3/12 | 7/12 | 9/11 | 12/12 | 12/12 | | 1/12 | 1/12 | 1/12 | 7/12 | 8/12 | |
| IGF1\_3 | 11/12 | 12/12 | 12/12 | 12/12 | 12/12 | | 11/12 | 12/12 | 12/12 | 12/12 | 12/12 | | 11/12 | 12/12 | 12/12 | 12/12 | 12/12 | |
| MSTN\_1 | 10/12 | 12/12 | 12/12 | 12/12 | 12/12 | | 9/12 | 12/12 | 12/12 | 12/12 | 12/12 | | 9/12 | 12/12 | 12/12 | 12/12 | 12/12 | |
| MSTN\_2 | 12/12 | 12/12 | 12/12 | 12/12 | 12/12 | | 11/12 | 10/12 | 12/12 | 12/12 | 12/12 | | 9/12 | 11/12 | 12/12 | 12/12 | 12/12 | |
| VEGFD\_1 | 11/12 | 11/12 | 12/12 | 12/12 | 12/12 | | 9/12 | 12/12 | 12/12 | 12/12 | 12/12 | | 11/12 | 12/12 | 12/12 | 12/12 | 12/12 | |
| VEGFD\_2 | 11/12 | 11/12 | 12/12 | 12/12 | 12/12 | | 4/12 | 10/12 | 9/12 | 12/12 | 12/12 | | 1/12 | 6/12 | 7/12 | 12/12 | 12/12 | |
| VEGFD\_3 | 11/12 | 11/12 | 12/12 | 12/12 | 12/12 | | 8/12 | 11/12 | 12/12 | 12/12 | 12/12 | | 6/12 | 9/12 | 12/12 | 12/12 | 12/12 | |
| VEGFA\_1 | 0/12 | 1/12 | 3/12 | 6/12 | 11/12 | | 0/12 | 0/12 | 0/11 | 8/12 | 12/12 | | 1/12 | 1/12 | 5/12 | 9/12 | 12/12 | |
| VEGFA\_2 | 11/12 | 12/12 | 12/12 | 12/12 | 12/12 | | 0/12 | 1/12 | 4/11 | 9/12 | 12/12 | | 3/12 | 2/12 | 5/12 | 6/12 | 12/12 | |
| VEGFA\_3 | 10/12 | 12/12 | 12/12 | 12/12 | 12/12 | | 2/12 | 4/12 | 6/11 | 9/12 | 12/12 | | 4/12 | 4/12 | 6/12 | 8/12 | 12/12 | |
| all |
| --- |
| > half of n |
| ≤ half of n |
| none |
Supplementary Figure 3. Assay test performances on serially diluted plasmid pools in gDNA background tested with the one-step (1 x PCR) and two-step (2 x PCR) PCR protocol. Shown are replicate numbers called positive in respect to the overall number of replicates (positive replicates / overall replicates). Green = all replicates, light yellow = more than half, dark yellow = half or less, red = none of the replicates were called positive.
9

## Slide 10
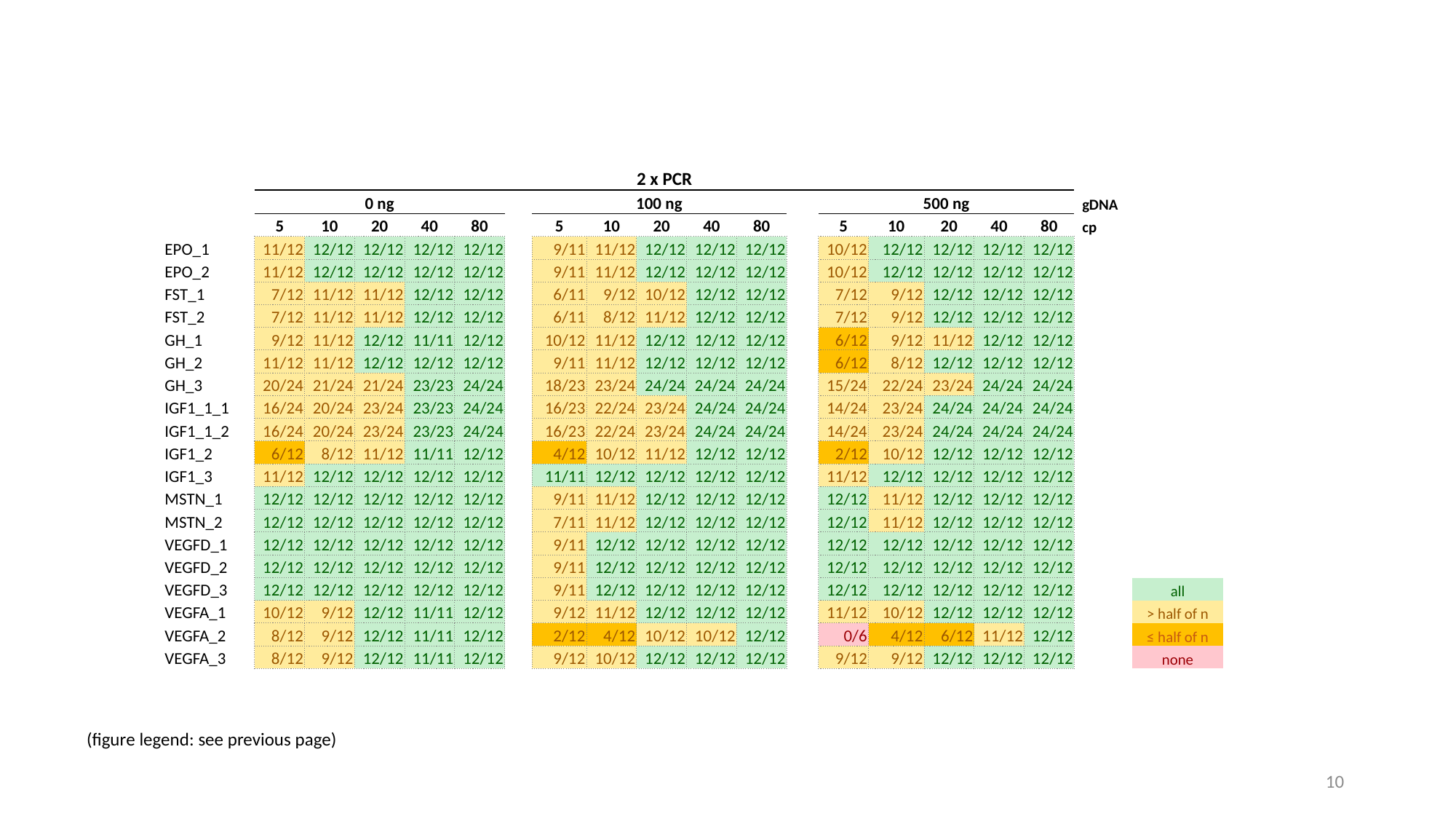

| | 2 x PCR | | | | | | | | | | | | | | | | | |
| --- | --- | --- | --- | --- | --- | --- | --- | --- | --- | --- | --- | --- | --- | --- | --- | --- | --- | --- |
| | 0 ng | | | | | | 100 ng | | | | | | 500 ng | | | | | gDNA |
| | 5 | 10 | 20 | 40 | 80 | | 5 | 10 | 20 | 40 | 80 | | 5 | 10 | 20 | 40 | 80 | cp |
| EPO\_1 | 11/12 | 12/12 | 12/12 | 12/12 | 12/12 | | 9/11 | 11/12 | 12/12 | 12/12 | 12/12 | | 10/12 | 12/12 | 12/12 | 12/12 | 12/12 | |
| EPO\_2 | 11/12 | 12/12 | 12/12 | 12/12 | 12/12 | | 9/11 | 11/12 | 12/12 | 12/12 | 12/12 | | 10/12 | 12/12 | 12/12 | 12/12 | 12/12 | |
| FST\_1 | 7/12 | 11/12 | 11/12 | 12/12 | 12/12 | | 6/11 | 9/12 | 10/12 | 12/12 | 12/12 | | 7/12 | 9/12 | 12/12 | 12/12 | 12/12 | |
| FST\_2 | 7/12 | 11/12 | 11/12 | 12/12 | 12/12 | | 6/11 | 8/12 | 11/12 | 12/12 | 12/12 | | 7/12 | 9/12 | 12/12 | 12/12 | 12/12 | |
| GH\_1 | 9/12 | 11/12 | 12/12 | 11/11 | 12/12 | | 10/12 | 11/12 | 12/12 | 12/12 | 12/12 | | 6/12 | 9/12 | 11/12 | 12/12 | 12/12 | |
| GH\_2 | 11/12 | 11/12 | 12/12 | 12/12 | 12/12 | | 9/11 | 11/12 | 12/12 | 12/12 | 12/12 | | 6/12 | 8/12 | 12/12 | 12/12 | 12/12 | |
| GH\_3 | 20/24 | 21/24 | 21/24 | 23/23 | 24/24 | | 18/23 | 23/24 | 24/24 | 24/24 | 24/24 | | 15/24 | 22/24 | 23/24 | 24/24 | 24/24 | |
| IGF1\_1\_1 | 16/24 | 20/24 | 23/24 | 23/23 | 24/24 | | 16/23 | 22/24 | 23/24 | 24/24 | 24/24 | | 14/24 | 23/24 | 24/24 | 24/24 | 24/24 | |
| IGF1\_1\_2 | 16/24 | 20/24 | 23/24 | 23/23 | 24/24 | | 16/23 | 22/24 | 23/24 | 24/24 | 24/24 | | 14/24 | 23/24 | 24/24 | 24/24 | 24/24 | |
| IGF1\_2 | 6/12 | 8/12 | 11/12 | 11/11 | 12/12 | | 4/12 | 10/12 | 11/12 | 12/12 | 12/12 | | 2/12 | 10/12 | 12/12 | 12/12 | 12/12 | |
| IGF1\_3 | 11/12 | 12/12 | 12/12 | 12/12 | 12/12 | | 11/11 | 12/12 | 12/12 | 12/12 | 12/12 | | 11/12 | 12/12 | 12/12 | 12/12 | 12/12 | |
| MSTN\_1 | 12/12 | 12/12 | 12/12 | 12/12 | 12/12 | | 9/11 | 11/12 | 12/12 | 12/12 | 12/12 | | 12/12 | 11/12 | 12/12 | 12/12 | 12/12 | |
| MSTN\_2 | 12/12 | 12/12 | 12/12 | 12/12 | 12/12 | | 7/11 | 11/12 | 12/12 | 12/12 | 12/12 | | 12/12 | 11/12 | 12/12 | 12/12 | 12/12 | |
| VEGFD\_1 | 12/12 | 12/12 | 12/12 | 12/12 | 12/12 | | 9/11 | 12/12 | 12/12 | 12/12 | 12/12 | | 12/12 | 12/12 | 12/12 | 12/12 | 12/12 | |
| VEGFD\_2 | 12/12 | 12/12 | 12/12 | 12/12 | 12/12 | | 9/11 | 12/12 | 12/12 | 12/12 | 12/12 | | 12/12 | 12/12 | 12/12 | 12/12 | 12/12 | |
| VEGFD\_3 | 12/12 | 12/12 | 12/12 | 12/12 | 12/12 | | 9/11 | 12/12 | 12/12 | 12/12 | 12/12 | | 12/12 | 12/12 | 12/12 | 12/12 | 12/12 | |
| VEGFA\_1 | 10/12 | 9/12 | 12/12 | 11/11 | 12/12 | | 9/12 | 11/12 | 12/12 | 12/12 | 12/12 | | 11/12 | 10/12 | 12/12 | 12/12 | 12/12 | |
| VEGFA\_2 | 8/12 | 9/12 | 12/12 | 11/11 | 12/12 | | 2/12 | 4/12 | 10/12 | 10/12 | 12/12 | | 0/6 | 4/12 | 6/12 | 11/12 | 12/12 | |
| VEGFA\_3 | 8/12 | 9/12 | 12/12 | 11/11 | 12/12 | | 9/12 | 10/12 | 12/12 | 12/12 | 12/12 | | 9/12 | 9/12 | 12/12 | 12/12 | 12/12 | |
| all |
| --- |
| > half of n |
| ≤ half of n |
| none |
(figure legend: see previous page)
10

## Slide 11
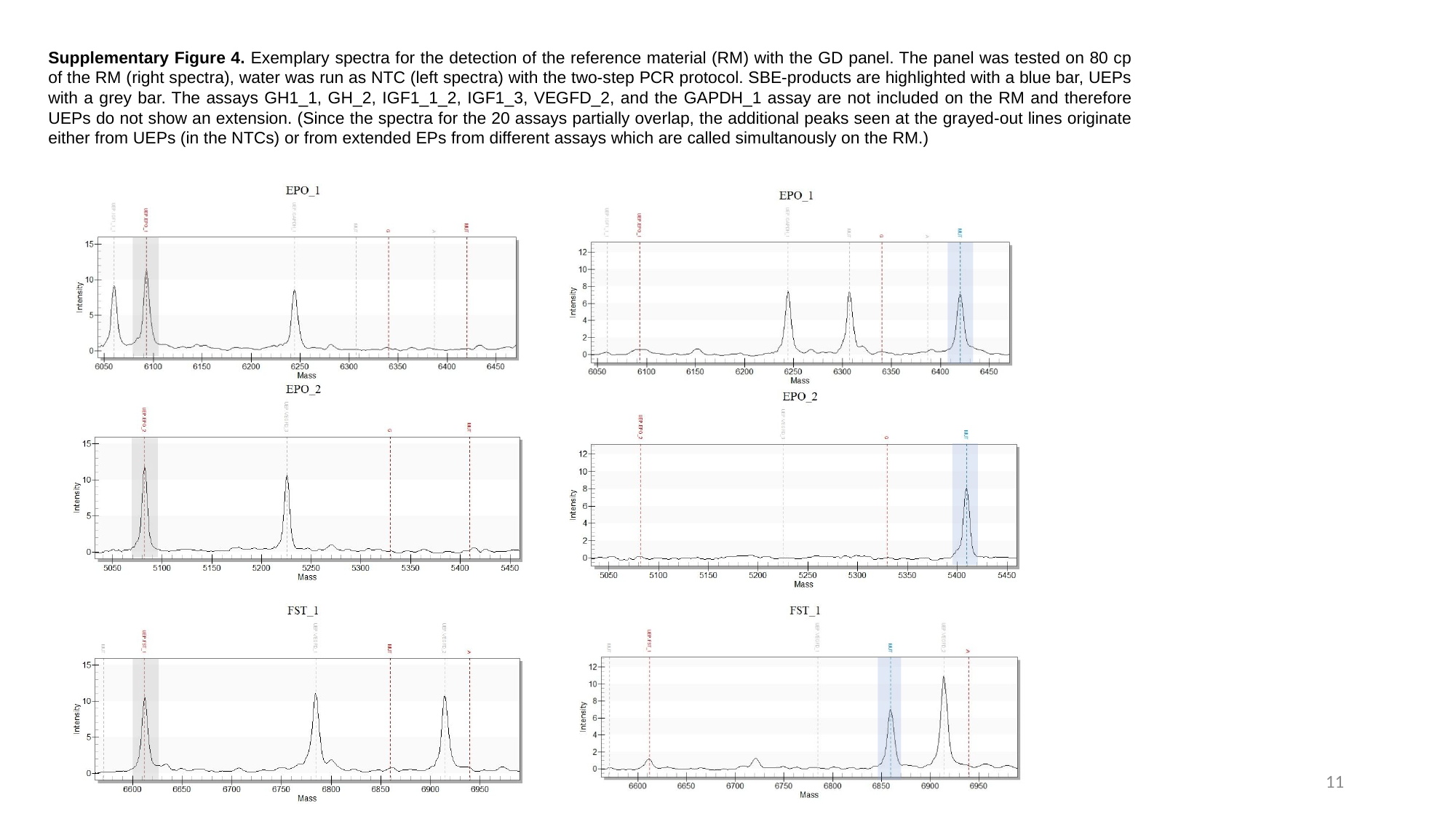

Supplementary Figure 4. Exemplary spectra for the detection of the reference material (RM) with the GD panel. The panel was tested on 80 cp of the RM (right spectra), water was run as NTC (left spectra) with the two-step PCR protocol. SBE-products are highlighted with a blue bar, UEPs with a grey bar. The assays GH1_1, GH_2, IGF1_1_2, IGF1_3, VEGFD_2, and the GAPDH_1 assay are not included on the RM and therefore UEPs do not show an extension. (Since the spectra for the 20 assays partially overlap, the additional peaks seen at the grayed-out lines originate either from UEPs (in the NTCs) or from extended EPs from different assays which are called simultanously on the RM.)
11

## Slide 12
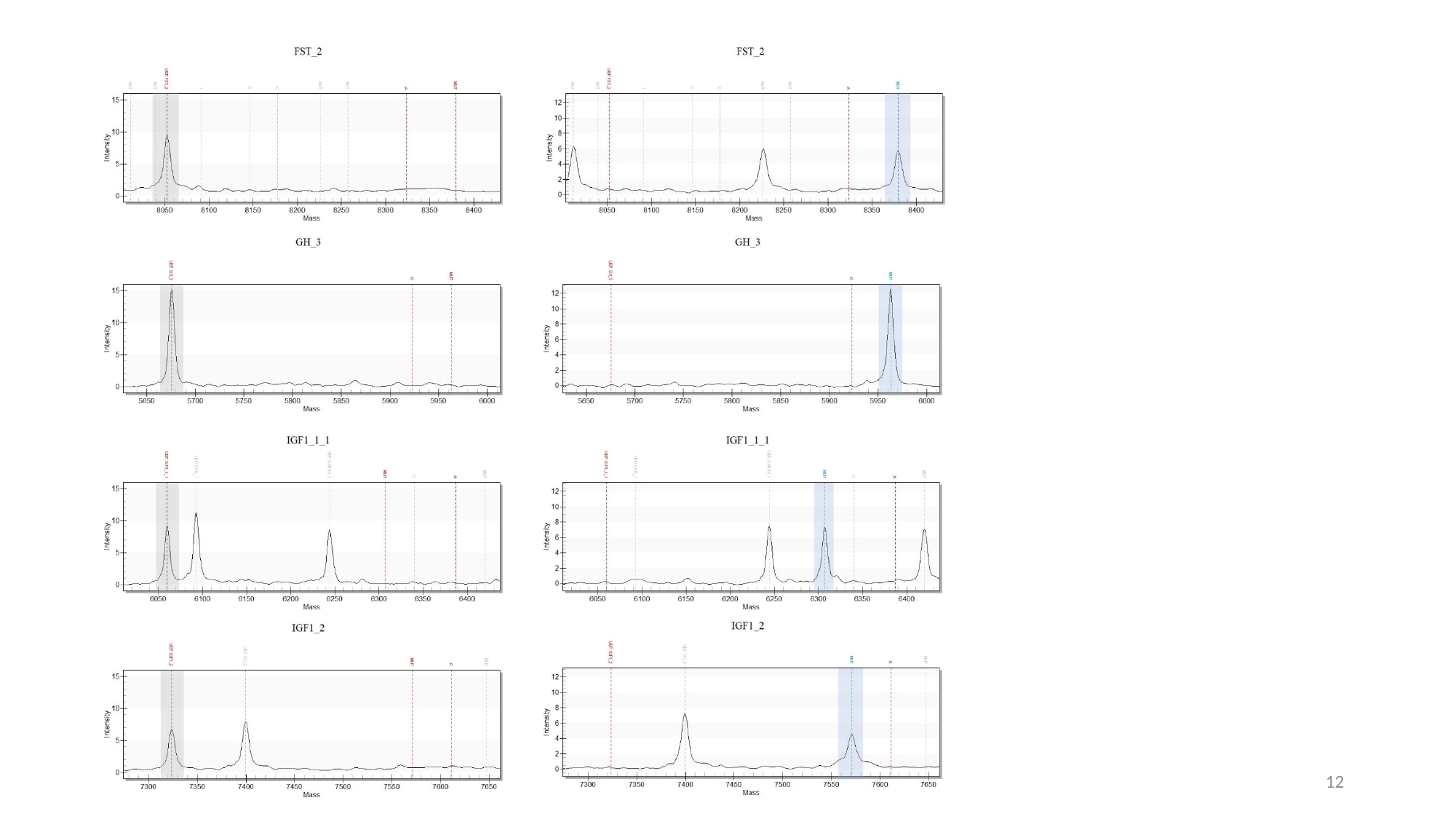

12

## Slide 13
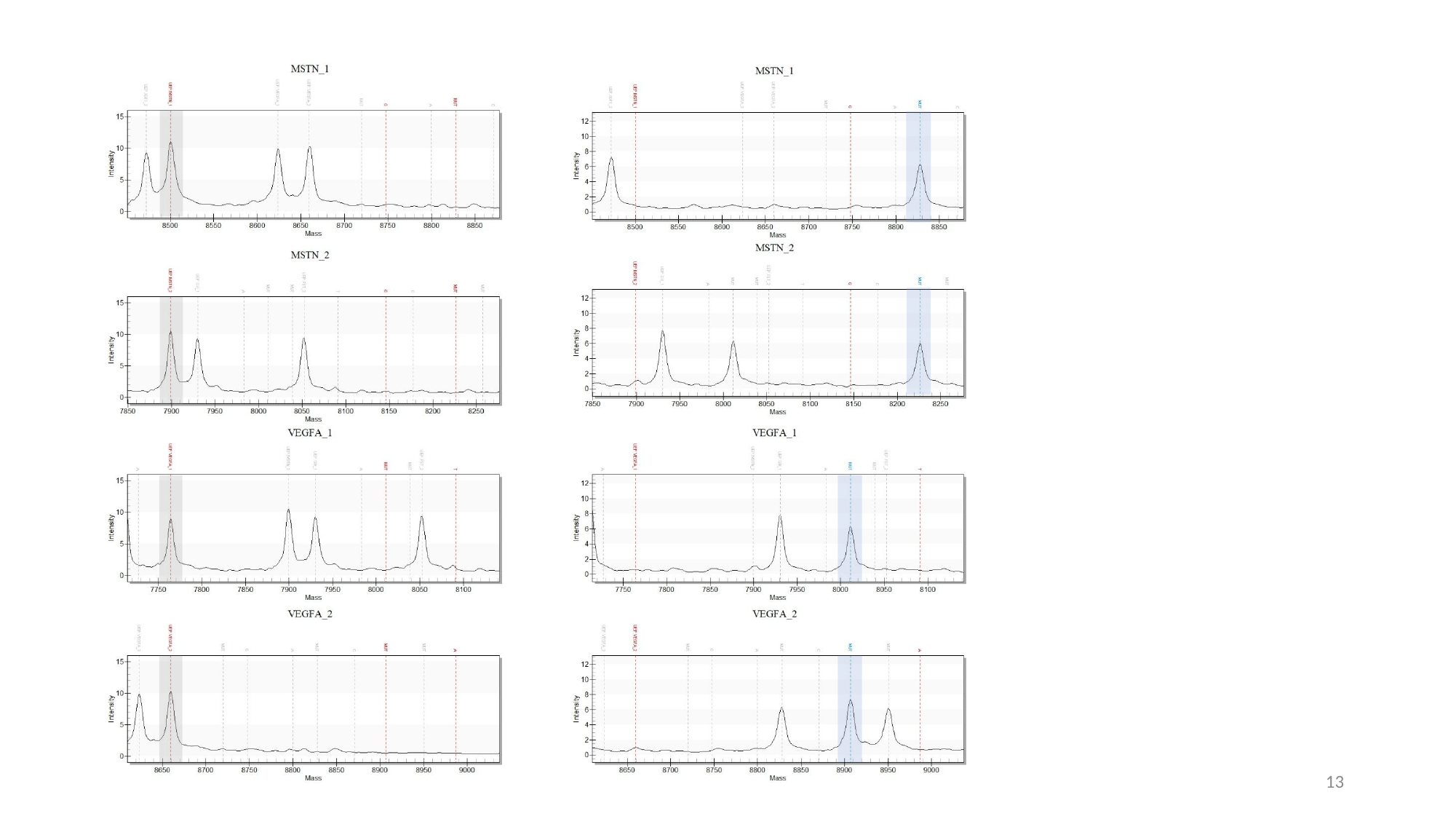

13

## Slide 14
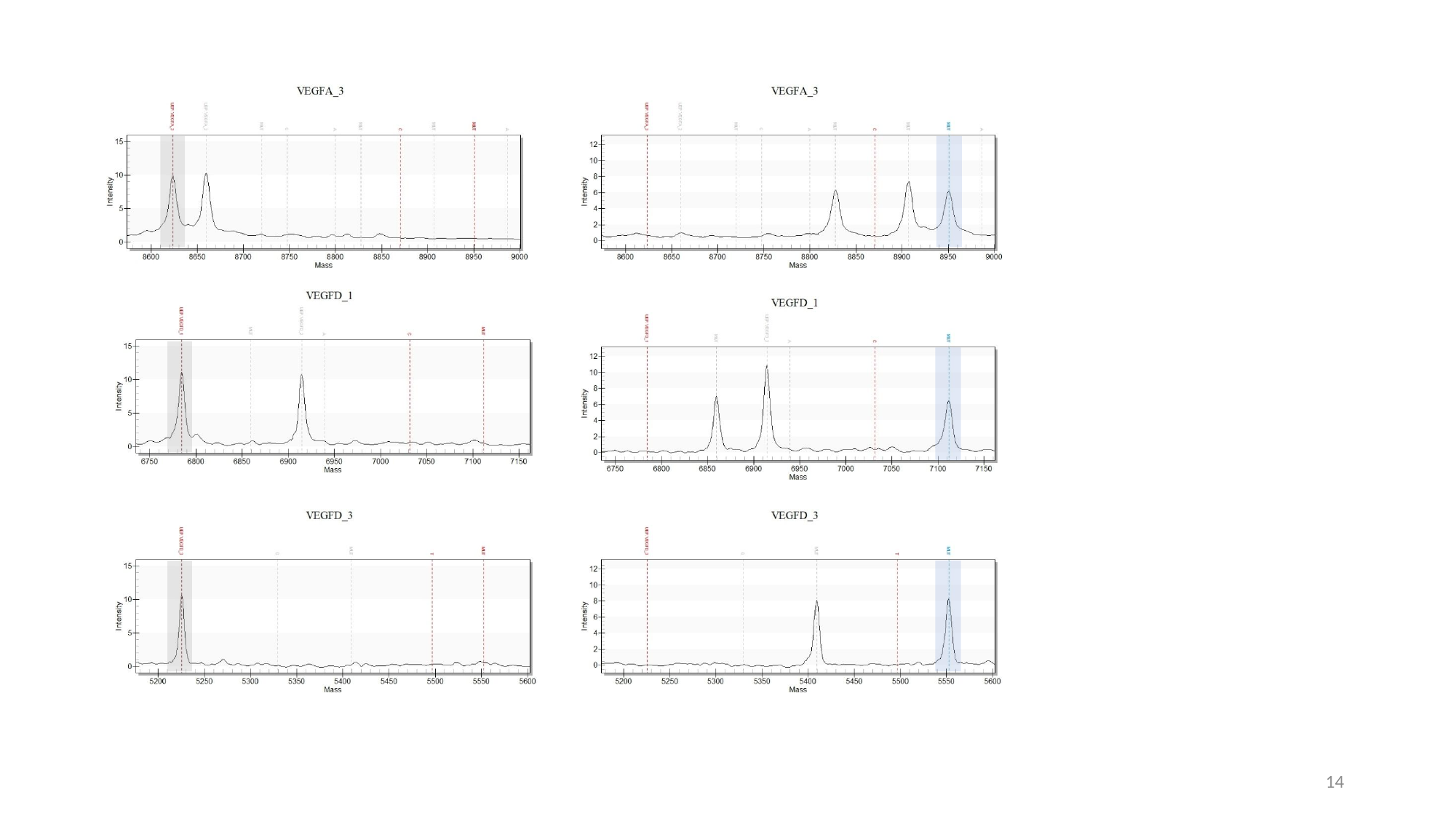

14

## Slide 15
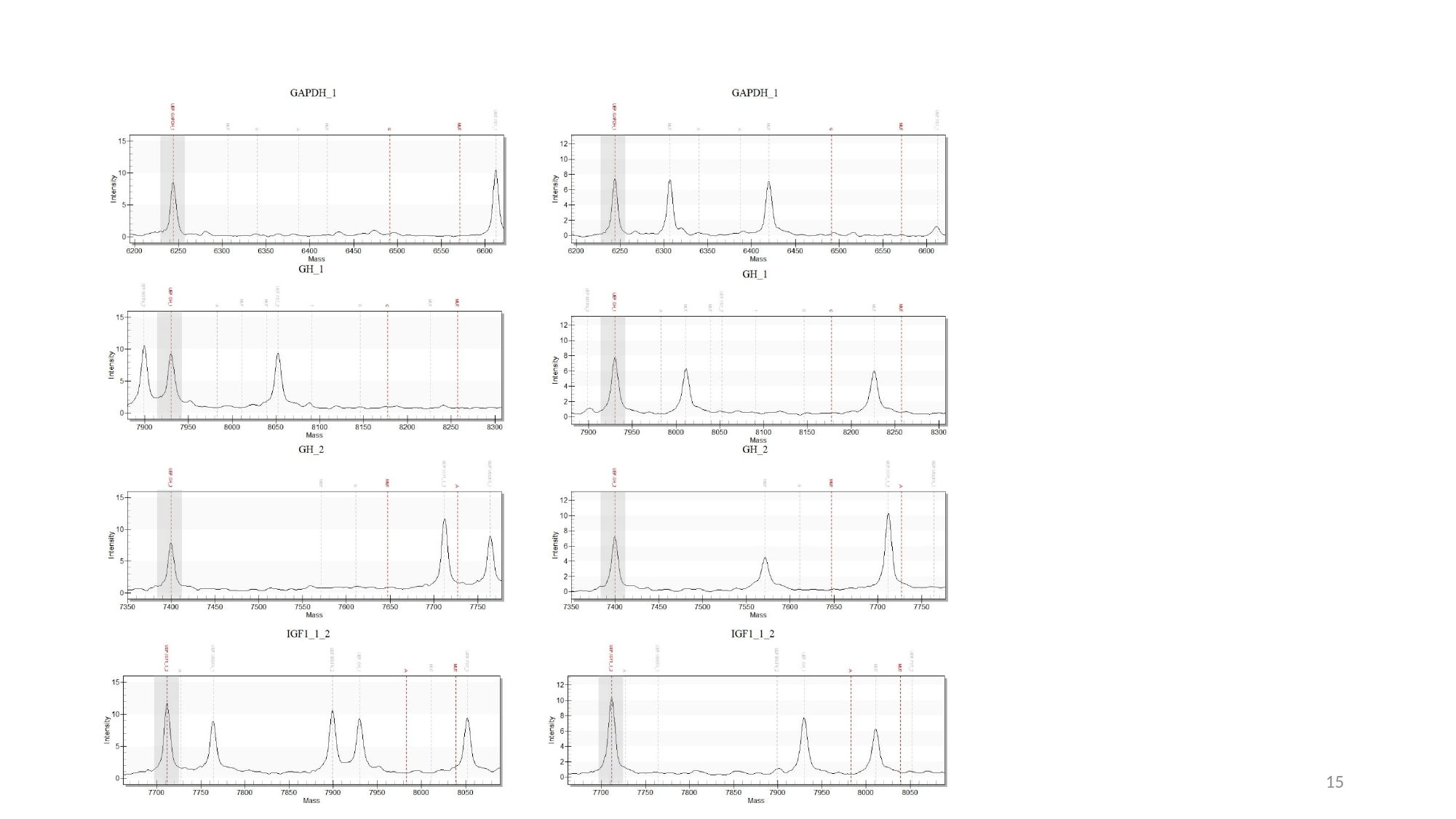

15

## Slide 16
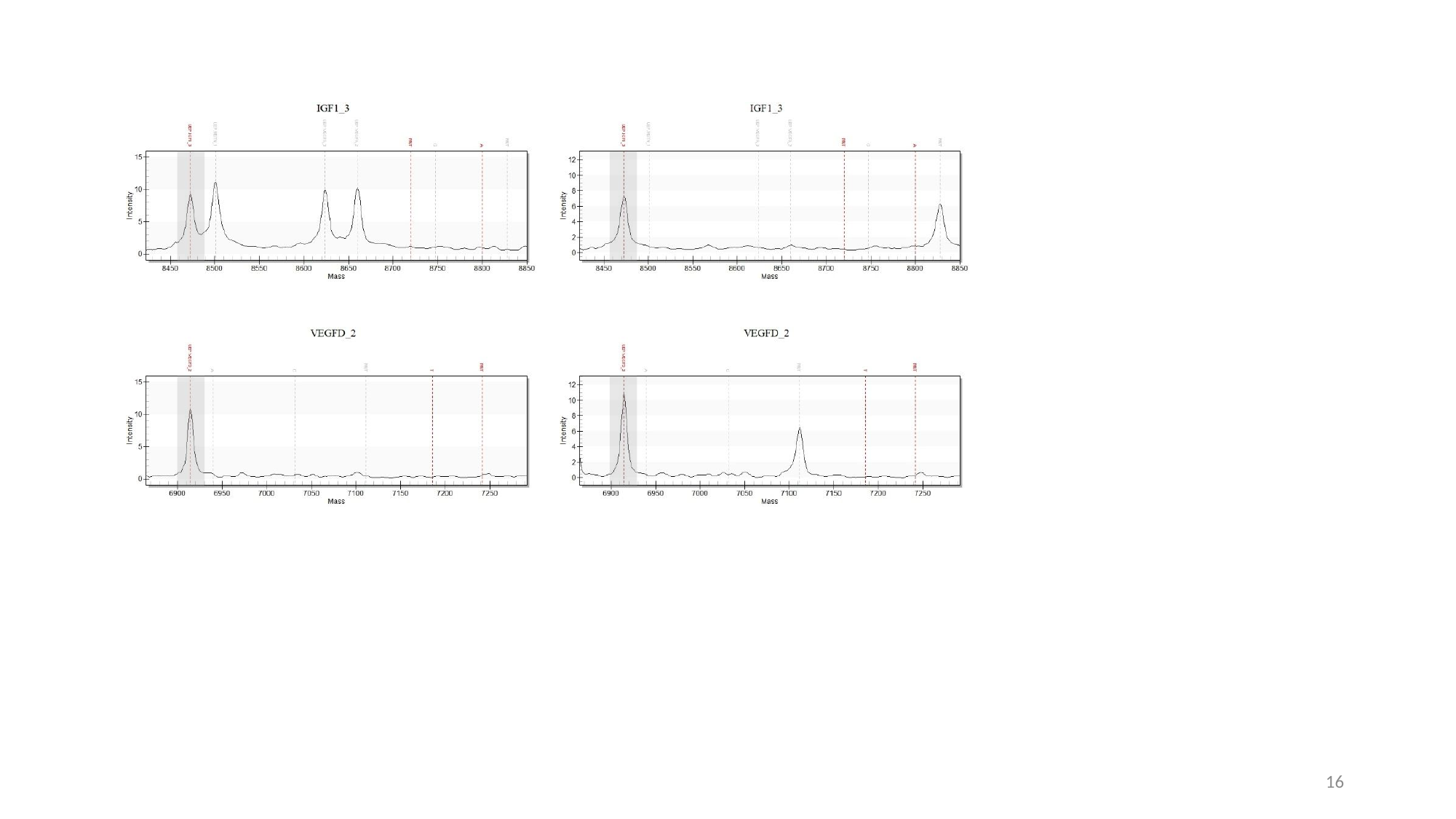

16

## Slide 17
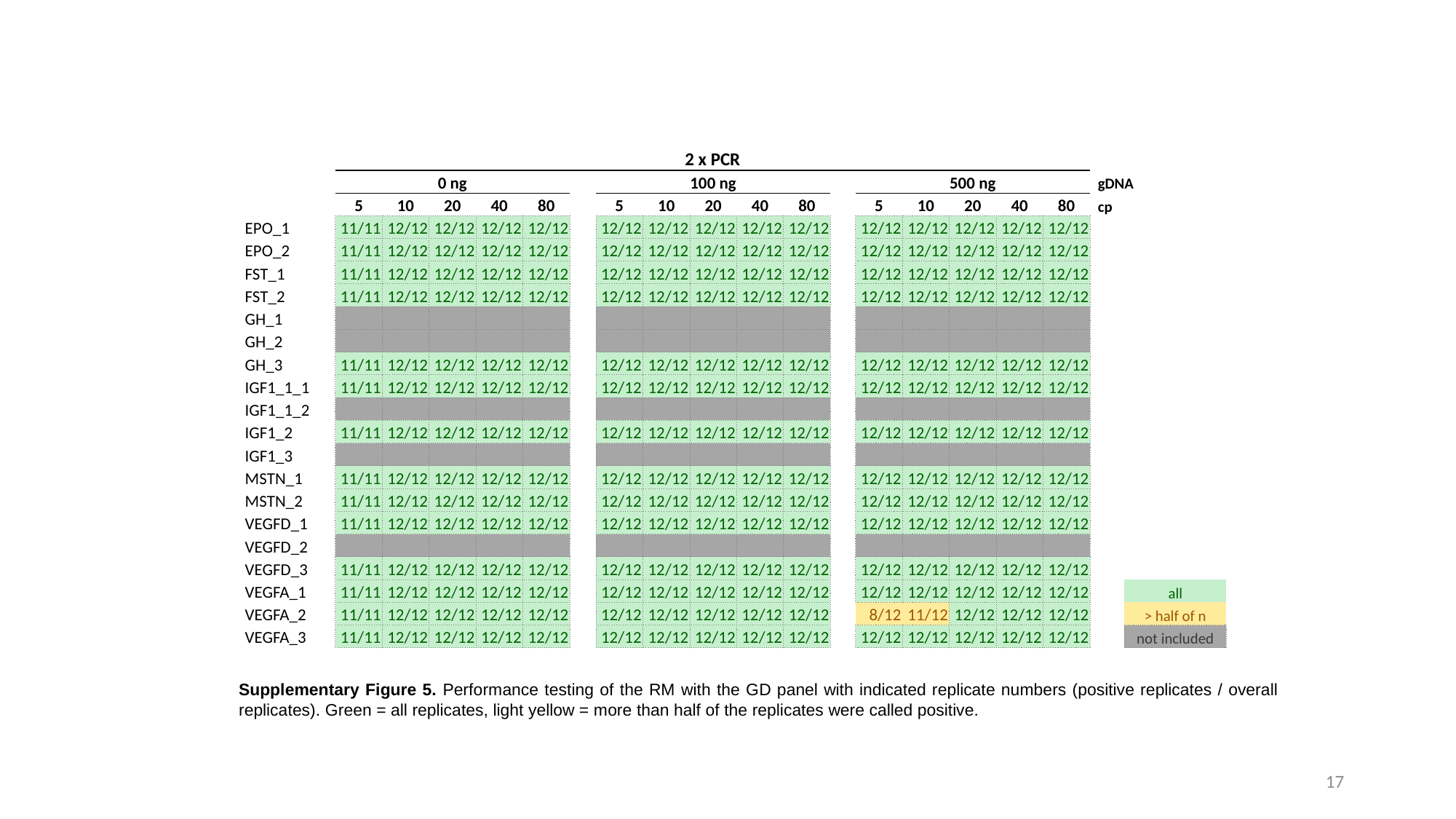

| | 2 x PCR | | | | | | | | | | | | | | | | | |
| --- | --- | --- | --- | --- | --- | --- | --- | --- | --- | --- | --- | --- | --- | --- | --- | --- | --- | --- |
| | 0 ng | | | | | | 100 ng | | | | | | 500 ng | | | | | gDNA |
| | 5 | 10 | 20 | 40 | 80 | | 5 | 10 | 20 | 40 | 80 | | 5 | 10 | 20 | 40 | 80 | cp |
| EPO\_1 | 11/11 | 12/12 | 12/12 | 12/12 | 12/12 | | 12/12 | 12/12 | 12/12 | 12/12 | 12/12 | | 12/12 | 12/12 | 12/12 | 12/12 | 12/12 | |
| EPO\_2 | 11/11 | 12/12 | 12/12 | 12/12 | 12/12 | | 12/12 | 12/12 | 12/12 | 12/12 | 12/12 | | 12/12 | 12/12 | 12/12 | 12/12 | 12/12 | |
| FST\_1 | 11/11 | 12/12 | 12/12 | 12/12 | 12/12 | | 12/12 | 12/12 | 12/12 | 12/12 | 12/12 | | 12/12 | 12/12 | 12/12 | 12/12 | 12/12 | |
| FST\_2 | 11/11 | 12/12 | 12/12 | 12/12 | 12/12 | | 12/12 | 12/12 | 12/12 | 12/12 | 12/12 | | 12/12 | 12/12 | 12/12 | 12/12 | 12/12 | |
| GH\_1 | | | | | | | | | | | | | | | | | | |
| GH\_2 | | | | | | | | | | | | | | | | | | |
| GH\_3 | 11/11 | 12/12 | 12/12 | 12/12 | 12/12 | | 12/12 | 12/12 | 12/12 | 12/12 | 12/12 | | 12/12 | 12/12 | 12/12 | 12/12 | 12/12 | |
| IGF1\_1\_1 | 11/11 | 12/12 | 12/12 | 12/12 | 12/12 | | 12/12 | 12/12 | 12/12 | 12/12 | 12/12 | | 12/12 | 12/12 | 12/12 | 12/12 | 12/12 | |
| IGF1\_1\_2 | | | | | | | | | | | | | | | | | | |
| IGF1\_2 | 11/11 | 12/12 | 12/12 | 12/12 | 12/12 | | 12/12 | 12/12 | 12/12 | 12/12 | 12/12 | | 12/12 | 12/12 | 12/12 | 12/12 | 12/12 | |
| IGF1\_3 | | | | | | | | | | | | | | | | | | |
| MSTN\_1 | 11/11 | 12/12 | 12/12 | 12/12 | 12/12 | | 12/12 | 12/12 | 12/12 | 12/12 | 12/12 | | 12/12 | 12/12 | 12/12 | 12/12 | 12/12 | |
| MSTN\_2 | 11/11 | 12/12 | 12/12 | 12/12 | 12/12 | | 12/12 | 12/12 | 12/12 | 12/12 | 12/12 | | 12/12 | 12/12 | 12/12 | 12/12 | 12/12 | |
| VEGFD\_1 | 11/11 | 12/12 | 12/12 | 12/12 | 12/12 | | 12/12 | 12/12 | 12/12 | 12/12 | 12/12 | | 12/12 | 12/12 | 12/12 | 12/12 | 12/12 | |
| VEGFD\_2 | | | | | | | | | | | | | | | | | | |
| VEGFD\_3 | 11/11 | 12/12 | 12/12 | 12/12 | 12/12 | | 12/12 | 12/12 | 12/12 | 12/12 | 12/12 | | 12/12 | 12/12 | 12/12 | 12/12 | 12/12 | |
| VEGFA\_1 | 11/11 | 12/12 | 12/12 | 12/12 | 12/12 | | 12/12 | 12/12 | 12/12 | 12/12 | 12/12 | | 12/12 | 12/12 | 12/12 | 12/12 | 12/12 | |
| VEGFA\_2 | 11/11 | 12/12 | 12/12 | 12/12 | 12/12 | | 12/12 | 12/12 | 12/12 | 12/12 | 12/12 | | 8/12 | 11/12 | 12/12 | 12/12 | 12/12 | |
| VEGFA\_3 | 11/11 | 12/12 | 12/12 | 12/12 | 12/12 | | 12/12 | 12/12 | 12/12 | 12/12 | 12/12 | | 12/12 | 12/12 | 12/12 | 12/12 | 12/12 | |
| all |
| --- |
| > half of n |
| not included |
Supplementary Figure 5. Performance testing of the RM with the GD panel with indicated replicate numbers (positive replicates / overall replicates). Green = all replicates, light yellow = more than half of the replicates were called positive.
17

## Slide 18
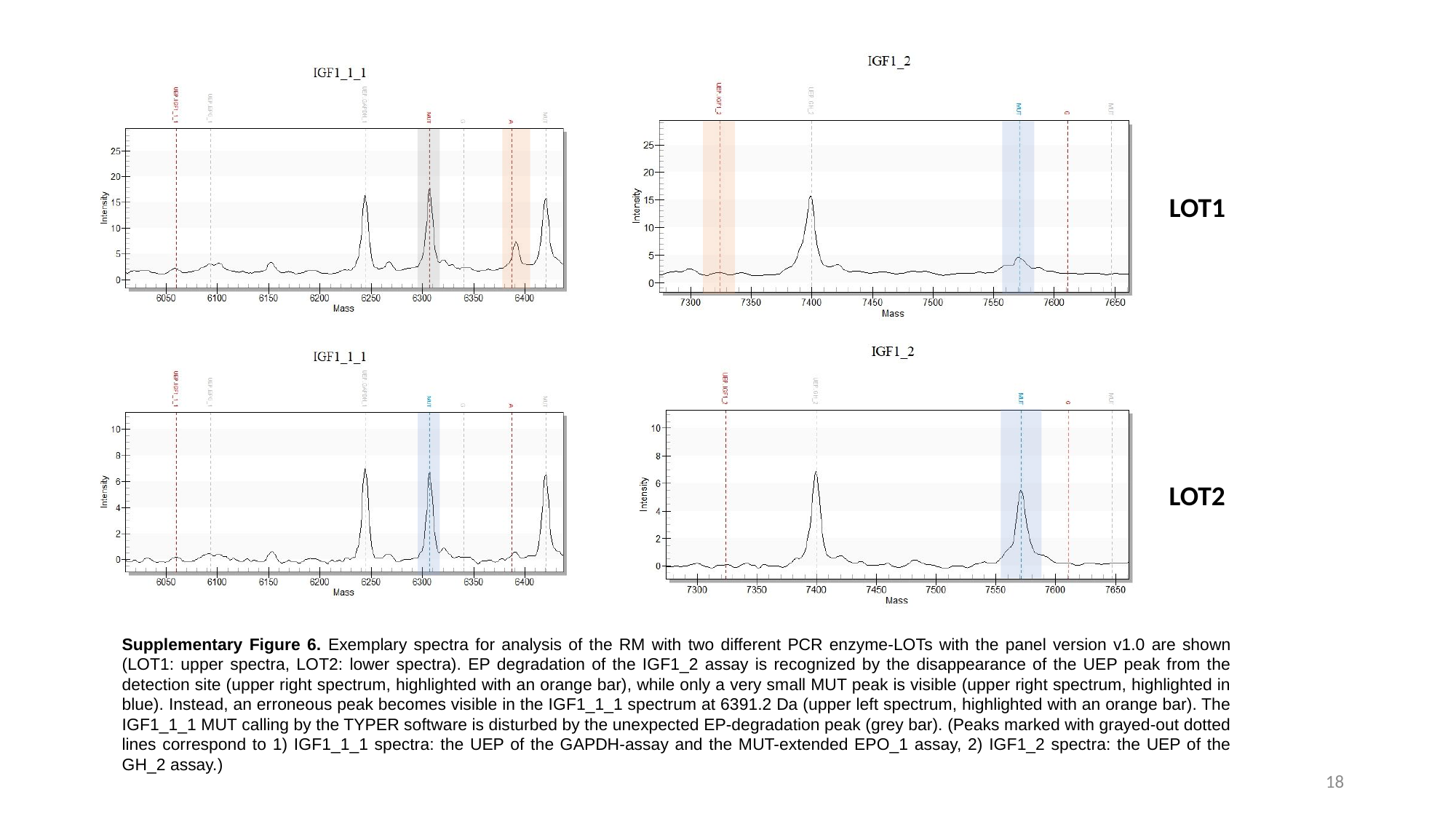

LOT1
LOT2
Supplementary Figure 6. Exemplary spectra for analysis of the RM with two different PCR enzyme-LOTs with the panel version v1.0 are shown (LOT1: upper spectra, LOT2: lower spectra). EP degradation of the IGF1_2 assay is recognized by the disappearance of the UEP peak from the detection site (upper right spectrum, highlighted with an orange bar), while only a very small MUT peak is visible (upper right spectrum, highlighted in blue). Instead, an erroneous peak becomes visible in the IGF1_1_1 spectrum at 6391.2 Da (upper left spectrum, highlighted with an orange bar). The IGF1_1_1 MUT calling by the TYPER software is disturbed by the unexpected EP-degradation peak (grey bar). (Peaks marked with grayed-out dotted lines correspond to 1) IGF1_1_1 spectra: the UEP of the GAPDH-assay and the MUT-extended EPO_1 assay, 2) IGF1_2 spectra: the UEP of the GH_2 assay.)
18

## Slide 19
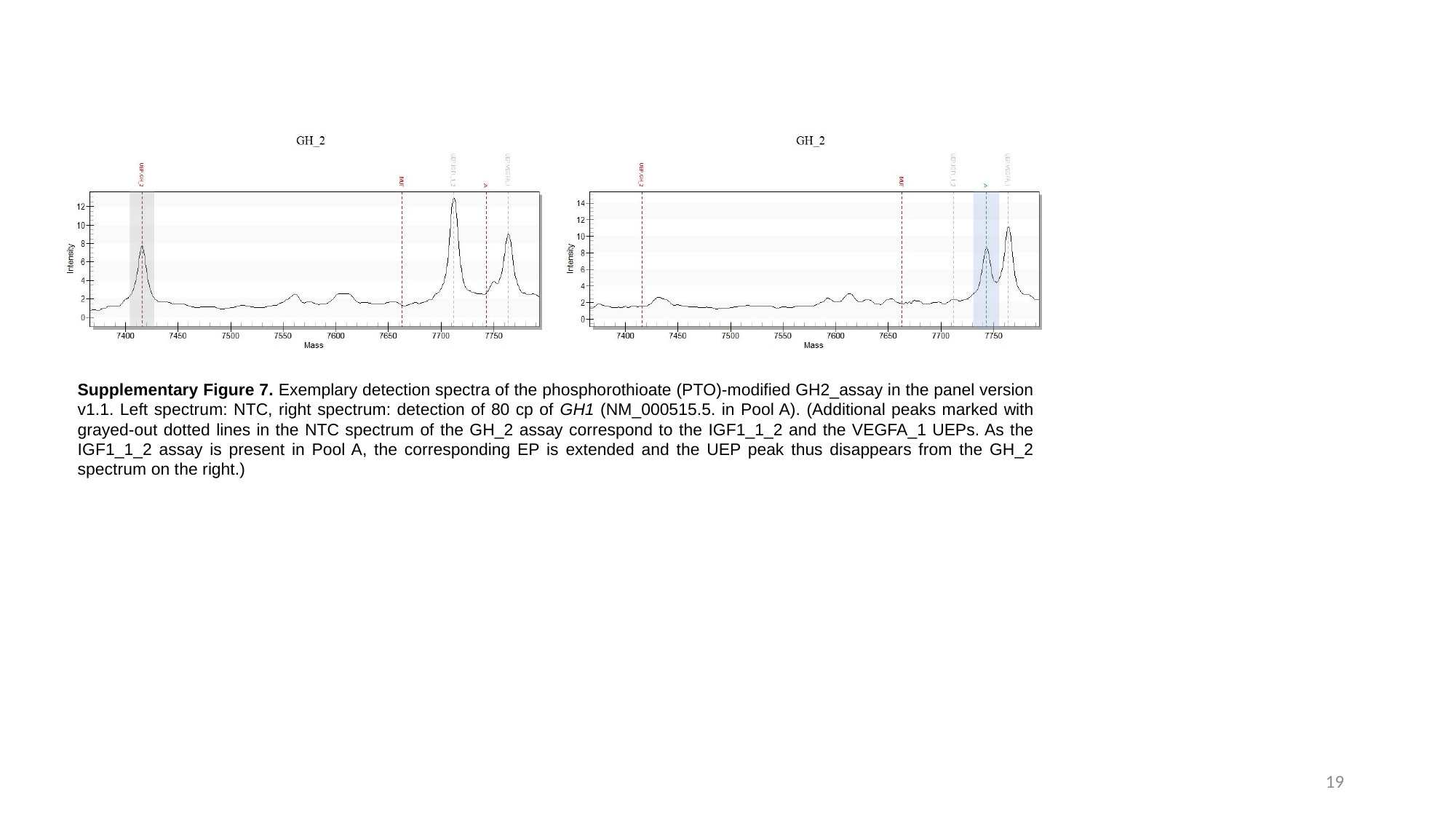

Supplementary Figure 7. Exemplary detection spectra of the phosphorothioate (PTO)-modified GH2_assay in the panel version v1.1. Left spectrum: NTC, right spectrum: detection of 80 cp of GH1 (NM_000515.5. in Pool A). (Additional peaks marked with grayed-out dotted lines in the NTC spectrum of the GH_2 assay correspond to the IGF1_1_2 and the VEGFA_1 UEPs. As the IGF1_1_2 assay is present in Pool A, the corresponding EP is extended and the UEP peak thus disappears from the GH_2 spectrum on the right.)
19

## Slide 20
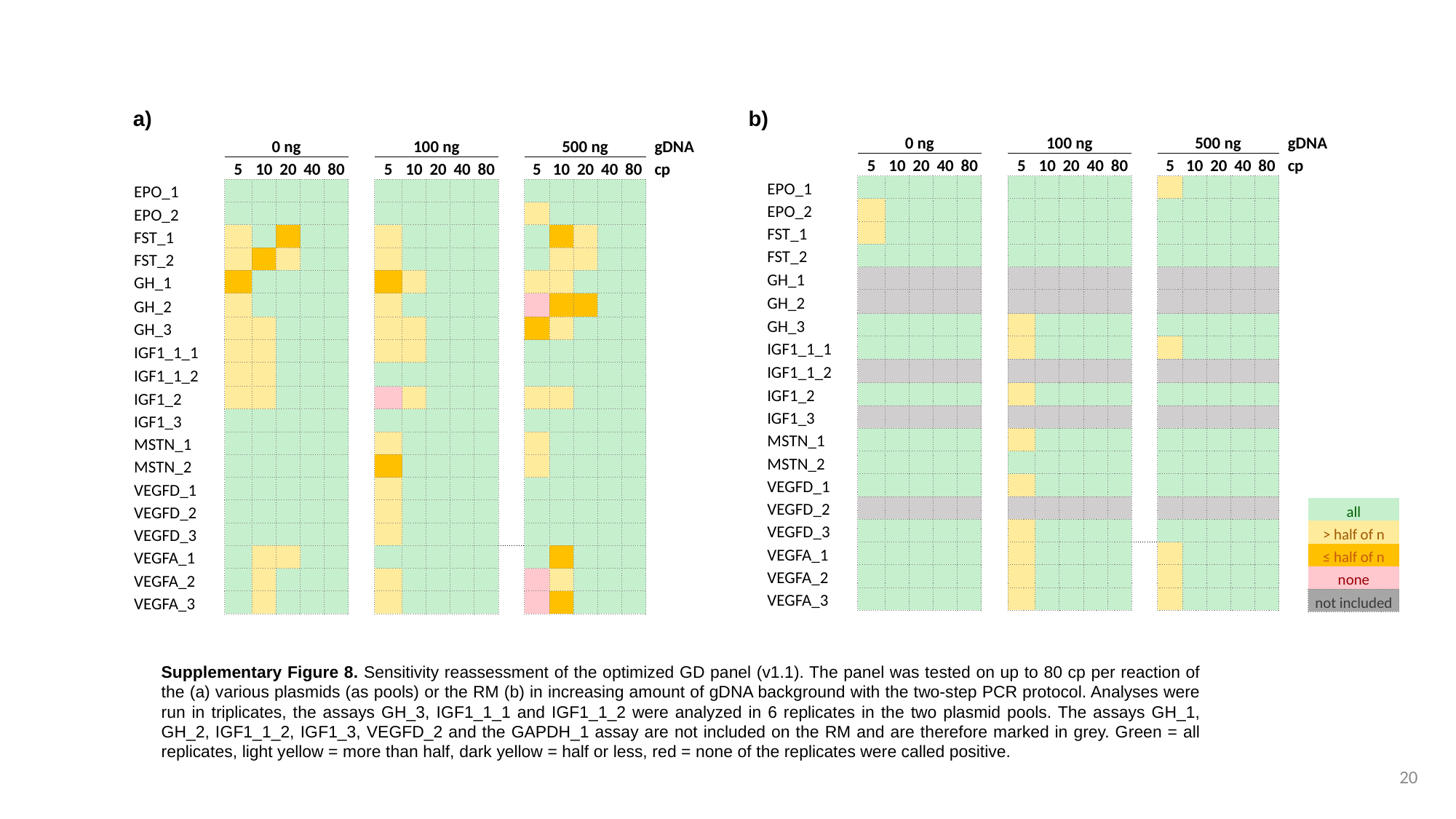

a)
b)
| | 0 ng | | | | | | 100 ng | | | | | | 500 ng | | | | | gDNA |
| --- | --- | --- | --- | --- | --- | --- | --- | --- | --- | --- | --- | --- | --- | --- | --- | --- | --- | --- |
| | 5 | 10 | 20 | 40 | 80 | | 5 | 10 | 20 | 40 | 80 | | 5 | 10 | 20 | 40 | 80 | cp |
| EPO\_1 | | | | | | | | | | | | | | | | | | |
| EPO\_2 | | | | | | | | | | | | | | | | | | |
| FST\_1 | | | | | | | | | | | | | | | | | | |
| FST\_2 | | | | | | | | | | | | | | | | | | |
| GH\_1 | | | | | | | | | | | | | | | | | | |
| GH\_2 | | | | | | | | | | | | | | | | | | |
| GH\_3 | | | | | | | | | | | | | | | | | | |
| IGF1\_1\_1 | | | | | | | | | | | | | | | | | | |
| IGF1\_1\_2 | | | | | | | | | | | | | | | | | | |
| IGF1\_2 | | | | | | | | | | | | | | | | | | |
| IGF1\_3 | | | | | | | | | | | | | | | | | | |
| MSTN\_1 | | | | | | | | | | | | | | | | | | |
| MSTN\_2 | | | | | | | | | | | | | | | | | | |
| VEGFD\_1 | | | | | | | | | | | | | | | | | | |
| VEGFD\_2 | | | | | | | | | | | | | | | | | | |
| VEGFD\_3 | | | | | | | | | | | | | | | | | | |
| VEGFA\_1 | | | | | | | | | | | | | | | | | | |
| VEGFA\_2 | | | | | | | | | | | | | | | | | | |
| VEGFA\_3 | | | | | | | | | | | | | | | | | | |
| | 0 ng | | | | | | 100 ng | | | | | | 500 ng | | | | | gDNA |
| --- | --- | --- | --- | --- | --- | --- | --- | --- | --- | --- | --- | --- | --- | --- | --- | --- | --- | --- |
| | 5 | 10 | 20 | 40 | 80 | | 5 | 10 | 20 | 40 | 80 | | 5 | 10 | 20 | 40 | 80 | cp |
| EPO\_1 | | | | | | | | | | | | | | | | | | |
| EPO\_2 | | | | | | | | | | | | | | | | | | |
| FST\_1 | | | | | | | | | | | | | | | | | | |
| FST\_2 | | | | | | | | | | | | | | | | | | |
| GH\_1 | | | | | | | | | | | | | | | | | | |
| GH\_2 | | | | | | | | | | | | | | | | | | |
| GH\_3 | | | | | | | | | | | | | | | | | | |
| IGF1\_1\_1 | | | | | | | | | | | | | | | | | | |
| IGF1\_1\_2 | | | | | | | | | | | | | | | | | | |
| IGF1\_2 | | | | | | | | | | | | | | | | | | |
| IGF1\_3 | | | | | | | | | | | | | | | | | | |
| MSTN\_1 | | | | | | | | | | | | | | | | | | |
| MSTN\_2 | | | | | | | | | | | | | | | | | | |
| VEGFD\_1 | | | | | | | | | | | | | | | | | | |
| VEGFD\_2 | | | | | | | | | | | | | | | | | | |
| VEGFD\_3 | | | | | | | | | | | | | | | | | | |
| VEGFA\_1 | | | | | | | | | | | | | | | | | | |
| VEGFA\_2 | | | | | | | | | | | | | | | | | | |
| VEGFA\_3 | | | | | | | | | | | | | | | | | | |
| all |
| --- |
| > half of n |
| ≤ half of n |
| none |
| not included |
Supplementary Figure 8. Sensitivity reassessment of the optimized GD panel (v1.1). The panel was tested on up to 80 cp per reaction of the (a) various plasmids (as pools) or the RM (b) in increasing amount of gDNA background with the two-step PCR protocol. Analyses were run in triplicates, the assays GH_3, IGF1_1_1 and IGF1_1_2 were analyzed in 6 replicates in the two plasmid pools. The assays GH_1, GH_2, IGF1_1_2, IGF1_3, VEGFD_2 and the GAPDH_1 assay are not included on the RM and are therefore marked in grey. Green = all replicates, light yellow = more than half, dark yellow = half or less, red = none of the replicates were called positive.
20

## Slide 21
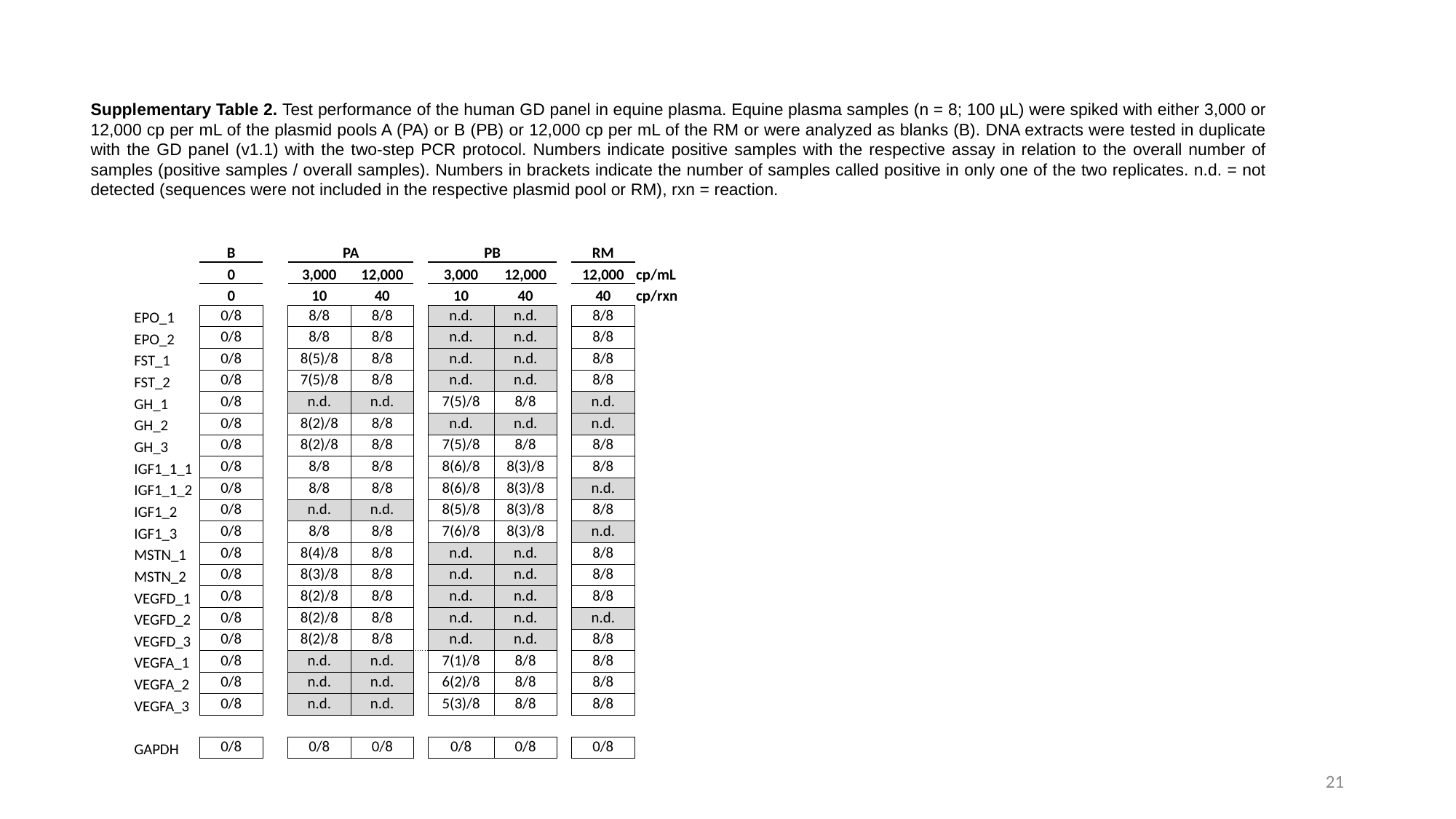

Supplementary Table 2. Test performance of the human GD panel in equine plasma. Equine plasma samples (n = 8; 100 µL) were spiked with either 3,000 or 12,000 cp per mL of the plasmid pools A (PA) or B (PB) or 12,000 cp per mL of the RM or were analyzed as blanks (B). DNA extracts were tested in duplicate with the GD panel (v1.1) with the two-step PCR protocol. Numbers indicate positive samples with the respective assay in relation to the overall number of samples (positive samples / overall samples). Numbers in brackets indicate the number of samples called positive in only one of the two replicates. n.d. = not detected (sequences were not included in the respective plasmid pool or RM), rxn = reaction.
| | B | | PA | | | PB | | | RM | |
| --- | --- | --- | --- | --- | --- | --- | --- | --- | --- | --- |
| | 0 | | 3,000 | 12,000 | | 3,000 | 12,000 | | 12,000 | cp/mL |
| | 0 | | 10 | 40 | | 10 | 40 | | 40 | cp/rxn |
| EPO\_1 | 0/8 | | 8/8 | 8/8 | | n.d. | n.d. | | 8/8 | |
| EPO\_2 | 0/8 | | 8/8 | 8/8 | | n.d. | n.d. | | 8/8 | |
| FST\_1 | 0/8 | | 8(5)/8 | 8/8 | | n.d. | n.d. | | 8/8 | |
| FST\_2 | 0/8 | | 7(5)/8 | 8/8 | | n.d. | n.d. | | 8/8 | |
| GH\_1 | 0/8 | | n.d. | n.d. | | 7(5)/8 | 8/8 | | n.d. | |
| GH\_2 | 0/8 | | 8(2)/8 | 8/8 | | n.d. | n.d. | | n.d. | |
| GH\_3 | 0/8 | | 8(2)/8 | 8/8 | | 7(5)/8 | 8/8 | | 8/8 | |
| IGF1\_1\_1 | 0/8 | | 8/8 | 8/8 | | 8(6)/8 | 8(3)/8 | | 8/8 | |
| IGF1\_1\_2 | 0/8 | | 8/8 | 8/8 | | 8(6)/8 | 8(3)/8 | | n.d. | |
| IGF1\_2 | 0/8 | | n.d. | n.d. | | 8(5)/8 | 8(3)/8 | | 8/8 | |
| IGF1\_3 | 0/8 | | 8/8 | 8/8 | | 7(6)/8 | 8(3)/8 | | n.d. | |
| MSTN\_1 | 0/8 | | 8(4)/8 | 8/8 | | n.d. | n.d. | | 8/8 | |
| MSTN\_2 | 0/8 | | 8(3)/8 | 8/8 | | n.d. | n.d. | | 8/8 | |
| VEGFD\_1 | 0/8 | | 8(2)/8 | 8/8 | | n.d. | n.d. | | 8/8 | |
| VEGFD\_2 | 0/8 | | 8(2)/8 | 8/8 | | n.d. | n.d. | | n.d. | |
| VEGFD\_3 | 0/8 | | 8(2)/8 | 8/8 | | n.d. | n.d. | | 8/8 | |
| VEGFA\_1 | 0/8 | | n.d. | n.d. | | 7(1)/8 | 8/8 | | 8/8 | |
| VEGFA\_2 | 0/8 | | n.d. | n.d. | | 6(2)/8 | 8/8 | | 8/8 | |
| VEGFA\_3 | 0/8 | | n.d. | n.d. | | 5(3)/8 | 8/8 | | 8/8 | |
| | | | | | | | | | | |
| GAPDH | 0/8 | | 0/8 | 0/8 | | 0/8 | 0/8 | | 0/8 | |
21
